# Revisiting the inhibitory potential of protein kinase inhibitors against NEK7 protein via comprehensive computational investigations

**DOI:** 10.1038/s41598-023-31499-7

**Published:** 2023-03-15

**Authors:** Syeda Abida Ejaz, Mubashir Aziz, Zeenat Zafar, Naveed Akhtar, Hanan A. Ogaly

**Affiliations:** 1 Department of Pharmaceutical Chemistry, Faculty of Pharmacy, The Islamia University of Bahawalpur, Bahawalpur, 63100 Pakistan; 2 Nistar Medical University Multan, Punjab, Pakistan; 3 Department of Pharmaceutics, Faculty of Pharmacy, The Islamia University of Bahawalpur, Bahawalpur, 63100 Pakistan; 4 Chemistry Department, College of Science, King Khalid University, Abha, 61421 Saudi Arabia; 5 Biochemistry and Molecular Biology Department, Faculty of Veterinary Medicine, Cairo University, Giza, 12211 Egypt

**Keywords:** Cancer, Computational biology and bioinformatics, Drug discovery

## Abstract

The NEK7 protein is required for spindle formation, cell division, and the activation of the NLRP3 inflammasome receptor. The aberrant expression of NEK7 has been implicated to the growth of metastasis and severe inflammatory conditions like rheumatoid arthritis, liver cirrhosis, and gout. An emergent target for the development of anti-cancer drugs is NEK7. In this context, the PubChem database was used to retrieve the 675 compound library and FDA-approved protein kinase inhibitors, which were then thoroughly examined via in-silico experiments. Computational studies investigated the binding orientation, electronic, and thermodynamic characteristics of drug candidates related to target protein. Drugs were investigated using density functional theory and molecular docking to find binding interactions with NEK7. Molecular dynamic simulations assessed interactions and stability of protein–ligand complex. DFT analyses showed that selected compounds maintained a significant amount of chemical reactivity in both liquid and gaseous states. Alectinib, Crizotinib, and compound 146476703 all displayed promising molecular interactions, according to molecular docking studies, with docking scores of − 32.76, − 30.54, and − 34.34 kJ/mol, respectively. Additionally, MD simulations determined the stability and dynamic characteristics of the complex over a 200 ns production run. The current study’s findings indicate that the drugs Alectinib, Crizotinib, and compound 146476703 can successfully inhibit the overexpression of the NEK7 protein. To discover more potent drugs against NEK7, it is recommended to synthesize the derivatives of Alectinib and Crizotinib and carry out additional in-vitro and in-vivo studies at the molecular level.

## Introduction

Cancer is a leading contributor to mortality worldwide, ranking as the second highest cause of death^1^. Despite notable advancements in the development of innovative cancer treatments, the demand for medical care continues to rise as our comprehension of cancer biology expands^2^. Cancer cells develop from an uncontrolled growth of cells that leads to genetic instability and abnormal cell proliferation^3^. Various therapeutic strategies have been devised to target cancer cells, but none have yet been successful in inhibiting the dysregulated enzymes that contribute to their growth^4^.

There are 518 protein kinases in the human genome, 478 of which have highly conserved sequences in their catalytic domains. The protein kinases that regulate cell cycle control include the NEK kinases, Aurora and Polo-Like Kinases (PLKs), as well as Cyclin-Dependent Kinases (CDKs). The K/E/D/D (Lys/Glu/Asp/Asp) signature was found in all of these protein kinases, which is required for the placement of adenosine triphosphate (ATP) and the stability of the active conformation. In the catalytic domain, the site where phosphorylation occurs and transforms the protein into its active form, kinase proteins possess a crucial ATP-binding site which triggers critical processes during cell division, such as cytokinesis, centrosome separation, and spindle formation^5^. While most protein kinases have been extensively studied as potential targets for anti-cancer drugs, the NEK kinases have received comparatively limited attention, except for NEK7. NEK7 has been identified as a significant player in the development of cancer and continues to be a promising target for the development of anticancer drugs. It is a unique member of the NEK family, with 85% structural similarity to NEK6 and a distinct role during cell division^6^. NEK7 has been shown to promote the formation of spindles and is involved in the regulation of intracellular protein trafficking, cell division, and inflammation^7^. Its aberrant expression has been linked to various types of cancer, including breast cancer, colon cancer, and non-small cell lung cancer^8,9^. 

The development of drugs targeting protein kinases holds great promise for anticancer therapy, as these drugs have been shown to possess anti-proliferative and anti-mitotic properties^10^. Currently, there are 53 FDA-approved drugs that inhibit protein kinases, including Alectinib and Crizotinib (carbonitrile and pyridine derivatives for breast cancer) and Erlotinib and Gefitinib (quinazoline derivatives for non-small cell lung cancer)^11^. Despite the advances in protein kinase-targeted anticancer drugs, the search for a selective inhibitor of NEK7 remains a challenge. To date, only Erlotinib and Gefitinib have been found to inhibit NEK7, but the extent of its inhibition and the mechanism of binding interaction are not yet known^12^. These findings underscore the ongoing need for the development of specific and effective drugs targeting NEK7.

**Figure 1:**
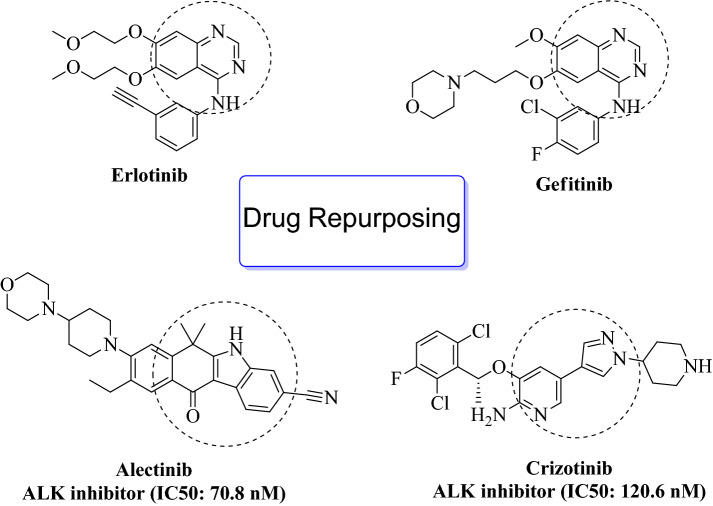
2D structures of protein kinase inhibitors (FDA approved drugs)^18^.

Instead of creating novel pharmacological molecules from scratch, computational repurposing of medications is a cutting-edge tool for the development of pharmaceuticals^13^. Presently, 30% of newly launched medications in US markets are the result of pharmacological repurposing. Aspirin, mifepristone, and topiramate are just a few examples of repurposed medications that are currently on the market and have been given FDA approval^14^. According to estimates, 6.7% of innovative anti-cancer medications developed between 2003 and 2011 were successful in getting FDA market approval after completing phase 1 trials^15,16^. This process took an average of 8.3 years. So, there is a need around the world for fast approval of effective cancer treatments, which can only be done by reusing drugs that have already been approved^17^. Figure 1 shows the 2D architecture of a protein kinase inhibitors.

**Figure 2:**
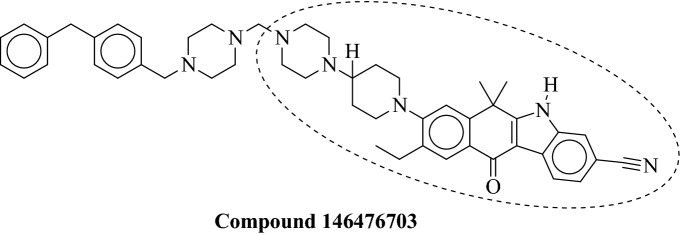
The hit molecule obtained through virtual screening against NEK7.

This study assessed the in-silico inhibitory capability of FDA-approved medications that were effective against different protein kinases. The initial goal of DFT investigations was to foretell molecular characteristics that sufficiently explained the chemical reactivity, bioactivity, and stability of drug candidates in gas and liquid phases^19,20^. After acquiring these improved drug structures, molecular docking studies with the NEK7 target were carried out to anticipate the free binding energies and binding orientation of a subset of medicines, which may help to comprehend the inhibitory mechanism. Then, using molecular dynamic (MD) simulations, the conformational flexibility and dynamic character of molecular interactions were determined. Additionally, 675 structurally related Alectinib analogues were virtually tested against the target protein after being downloaded from the PubChem database. The compound exhibited a higher docking score than standard Alectinib and was considered a hit and subjected to in-silico ADMET prediction and MD simulation studies. Figure 2 shows the identified hit obtained via virtual screening against NEK7.

## Results and discussion

### Density functional theory calculations

In the present work, structural geometry optimizations, the energetic parameters, FMOs analysis, and global reactivity descriptors of Alectinib, Crizotinib, Erlotinib, and Gefitinib were calculated using Gaussian 09W. Gauss View 6 was used for the visualization of output files. Investigations were conducted using the DFT/B3LYP functional and the SVP basis set.

#### Optimized geometries

**Figure 3:**
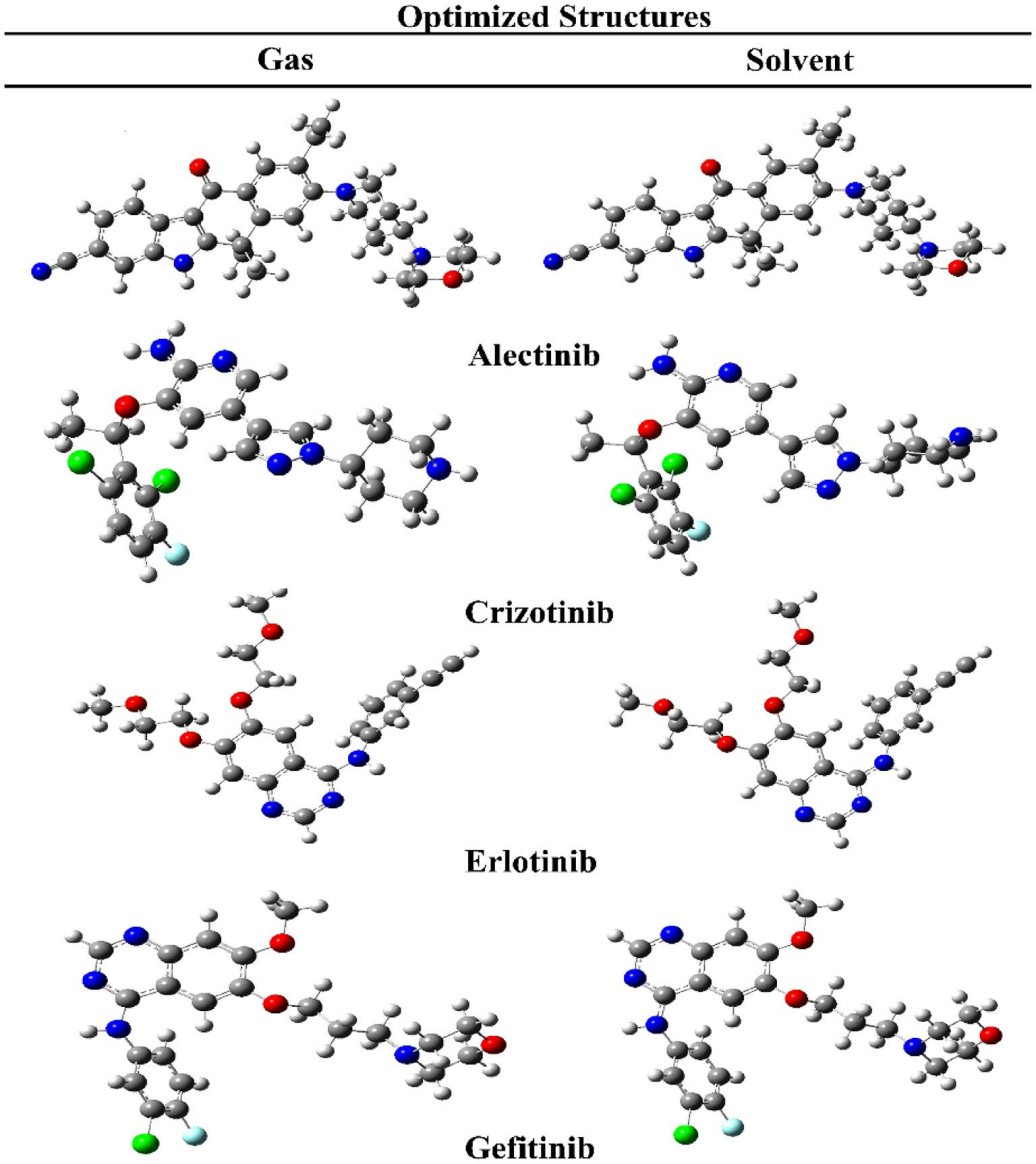
Optimized structures for FDA approved drugs (solvent; Water).

In two phases (gas and water), the geometry of selected FDA drugs was completely optimized. After optimizing the geometry, no imaginary frequencies were found, indicating that the current geometries are real local minima. It was discovered that Alectinib and Erlotinib have gas phase optimization energies of − 1532.70 and − 1316.84 Hz, respectively, showing their space stability. Only Alectinib showed a high polarizability value in both phases, i.e., the gas and the solvent phases (370.94 and 496.71 a.u respectively). In addition, the comprehensive geometric parameters of optimized drugs is provided in Supplementary Files (Tables S4–S7). The optimized structures of FDA drugs are shown in Fig. 3.

#### Frontier molecular orbital analysis (FMOs)

The FMOs are necessary because they affect the molecule’s reactivity and stability. The outermost electrons actively engage in the interaction between the ligand and the target protein. The highest occupied molecular orbitals (HOMO) and lowest unoccupied molecular orbitals (LUMO) provide sufficient insight into the chemical reactivity of the compounds. The HOMO reflects a compound’s capacity to give electrons, while the LUMO represents its ability to take or remove electrons. A very small energy gap E (ELUMO–EHOMO) between HOMO and LUMO indicates effective charge transfer and increases molecule polarizability^21^. Molecules with large E gaps (ELUMO–EHOMO) are non-polarizable and considered the least reactive chemical entities. The computed values for the HOMO/LUMO and E gap (ELUMO–EHOMO) are shown in Table 1.

**Table 1:** Optimization energy and frontier molecular orbitals analysis of FDA approved drugs.

Code	Gas			Solvent (water)					
	Optimization energy (hartree)	Polarizability a.u (α)	Dipole Moment (debye)	Optimization energy (hartree)	Polarizability a.u (α)	Dipole moment (debye)			
Alectinib	− 1532.701	370.944	9.736	− 1532.728	496.718	13.188			
Crizotinib	− 2182.274	271.421	1.756	− 2182.295	356.638	1.727			
Erlotinib	− 1316.847	281.805	5.084	− 1316.866	364.693	6.820			
Gefitinib	− 1857.041	289.168	4.220	− 1857.062	375.457	4.982			

Compound		E_HOMO_ (eV)	E_LUMO_ (eV)	∆E_gap_ (eV)	Potential ionization I (eV)	Affinity A (eV)	Electron donating power (ω−)	Electron accepting Power (ω+)	Electro philicity (Δω±)
Alectinib	Gas	− 0.195	− 0.057	0.138	0.195	0.057	0.187	0.061	0.248
	Sol	− 0.188	− 0.059	0.128	0.188	0.059	0.187	0.061	0.248
Crizotinib	Gas	− 0.191	− 0.046	0.144	0.191	0.046	0.167	0.048	0.215
	Sol	− 0.198	− 0.045	0.152	0.198	0.045	0.167	0.048	0.215
Erlotinib	Gas	− 0.211	− 0.055	0.155	0.211	0.055	0.192	0.058	0.250
	Sol	− 0.217	− 0.063	0.153	0.217	0.063	0.196	0.060	0.256
Gefitinib	Gas	− 0.195	− 0.06	0.128	0.195	0.062	0.204	0.071	0.275
	sol	− 0.196	− 0.063	0.133	0.196	0.063	0.244	0.099	0.343

The energy band gap between (ELUMO–EHOMO) for compound Alectinib and crizotinib was 0.138 eV and 0.144 eV in gas whereas it was 0.128 eV and 0.152 eV in solvent phase, respectively. Whereas, energy gap for Erlotinib and Gefitinib was 0.155 and 0.128 in gas phase and 0.153 and 0.133 eV in the solvent phase, respectively. It was discovered that a small energy gap corresponded to the compound’s increased reactivity. In addition, two important parameters i.e., chemical softness and hardness is measure of reactivity of a compound^22^. Compounds with a large ΔE (ELUMO–EHOMO) energy gap refer to the molecule’s least reactivity and more stability, while compounds with a small energy gap are often more reactive. In the solvent phase, Alectinib exhibited a chemical softness value of 7.77 whereas Crizotinib exhibited a softness indices of 6.55, indicating that Alectinib is highly reactive drug than Crizotinib. Likewise, Erlotinib and Gefitinib exhibited good value for chemical softness in solvent phase i.e., 5.50 and 7.50 respectively. They are tending to be more polarizable compounds. The Koopmans’s theorem was used to calculate the electron affinity and ionization energies of selected drugs^23^. The calculated values for hardness and softness are given in Table 2.

$${\text{I }} = - {\text{EHOMO}}; {\text{A}} = - {\text{ELUMO}}{.}$$

whereas other chemical parameters were calculated as follows;

**Table 2:** Global reactivity descriptors.

Compound		Chemical hardness (η)	Chemical potential (μ)	Electrophilicity index (ω)	Chemical softness (S)	Electronegativity (X)
Alectinib	Gas	0.069	− 0.127	0.116	7.223	0.127
	Sol*	0.064	− 0.124	0.119	7.772	0.124
Crizotinib	Gas	0.072	− 0.119	0.098	6.940	0.119
	Sol	0.076	− 0.122	0.098	6.550	0.122
Erlotinib	Gas	0.078	− 0.134	0.115	6.439	0.134
	Sol	0.077	− 0.141	0.128	5.502	0.141
Gefitinib	Gas	0.064	− 0.129	0.129	7.754	0.129
	Sol	0.067	− 0.130	0.126	7.506	0.130

*Sol = water.

Electrophilicity index: ω = µ/2η, chemical hardness: η = 1/2 (ELUMO − EHOMO); chemical potential: µ =  − χ; chemical softness: S = 1/2η; electronegativity =  − 1/2 (ELUMO + EHOMO).

**Figure 4:**
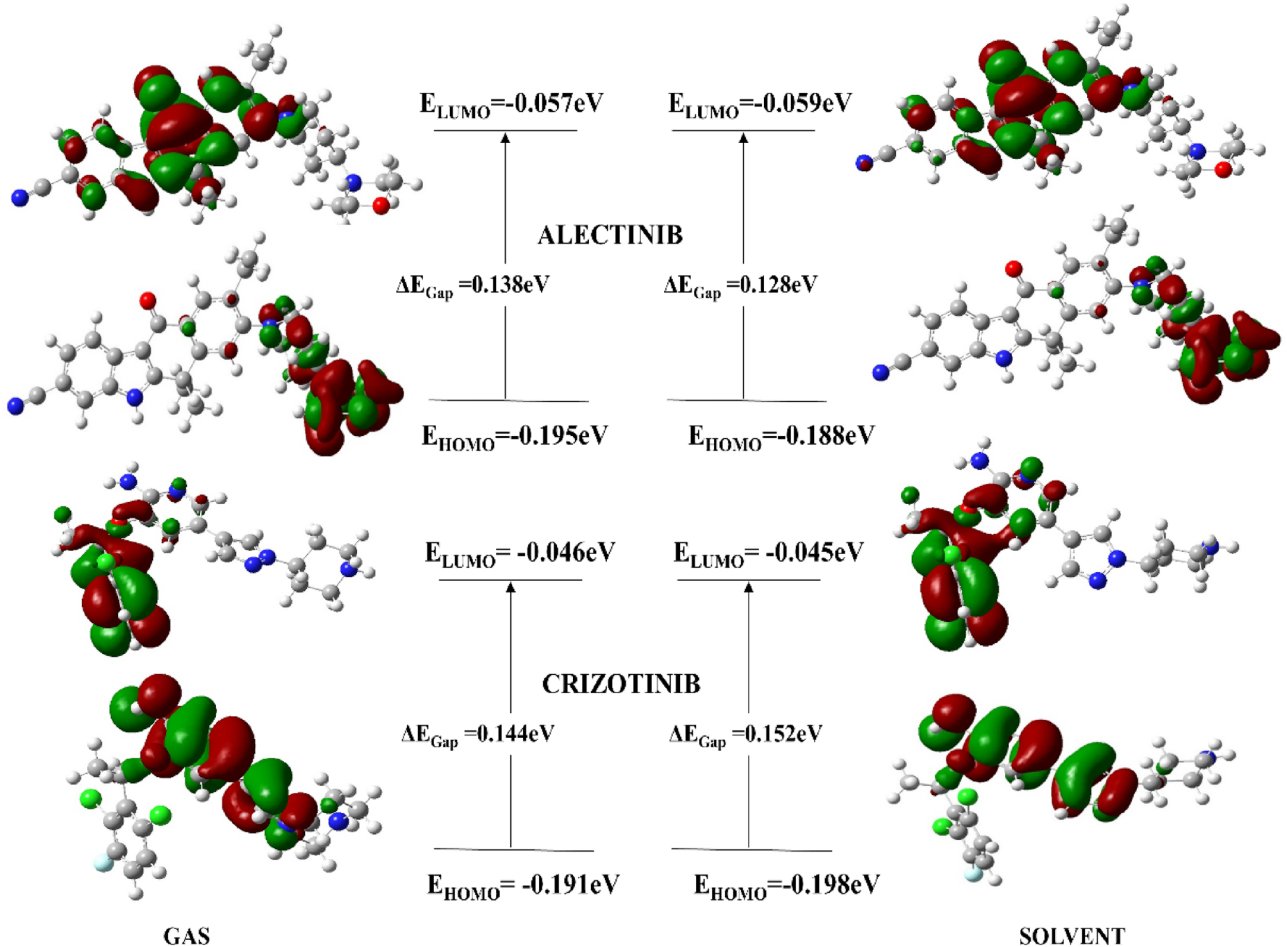
HOMO/LUMO orbitals of Alectinib and Crizotinib.

It is necessary to determine the electron density of the lowest unoccupied molecular orbital and the highest occupied molecular orbital because valance electrons are involved in the interaction and chemical reactivity of the compound. The current study has investigated the electron density of FDA drugs. The electron density of HOMO orbital in the gas phase for Alectinib was found to be confined to the morpholine and the piperdinyl ring, whereas the electron density of LUMO orbitals was restricted to the carbo-nitrile and the benzocarbazole moiety. The HOMO and LUMO electron densities of Alectinib in the solvent phase were substantially identical to the gas phase, albeit it was more limited toward the carbonitrile ring. Similarly, the electron density of HOMO orbitals of the Crizotinib molecule were confined to the pyrazole and pyridine rings, while the LUMO orbitals were confined to the trifluorophenyl ring. The FMOs of both drugs are shown in Fig. 4.

**Figure 5:**
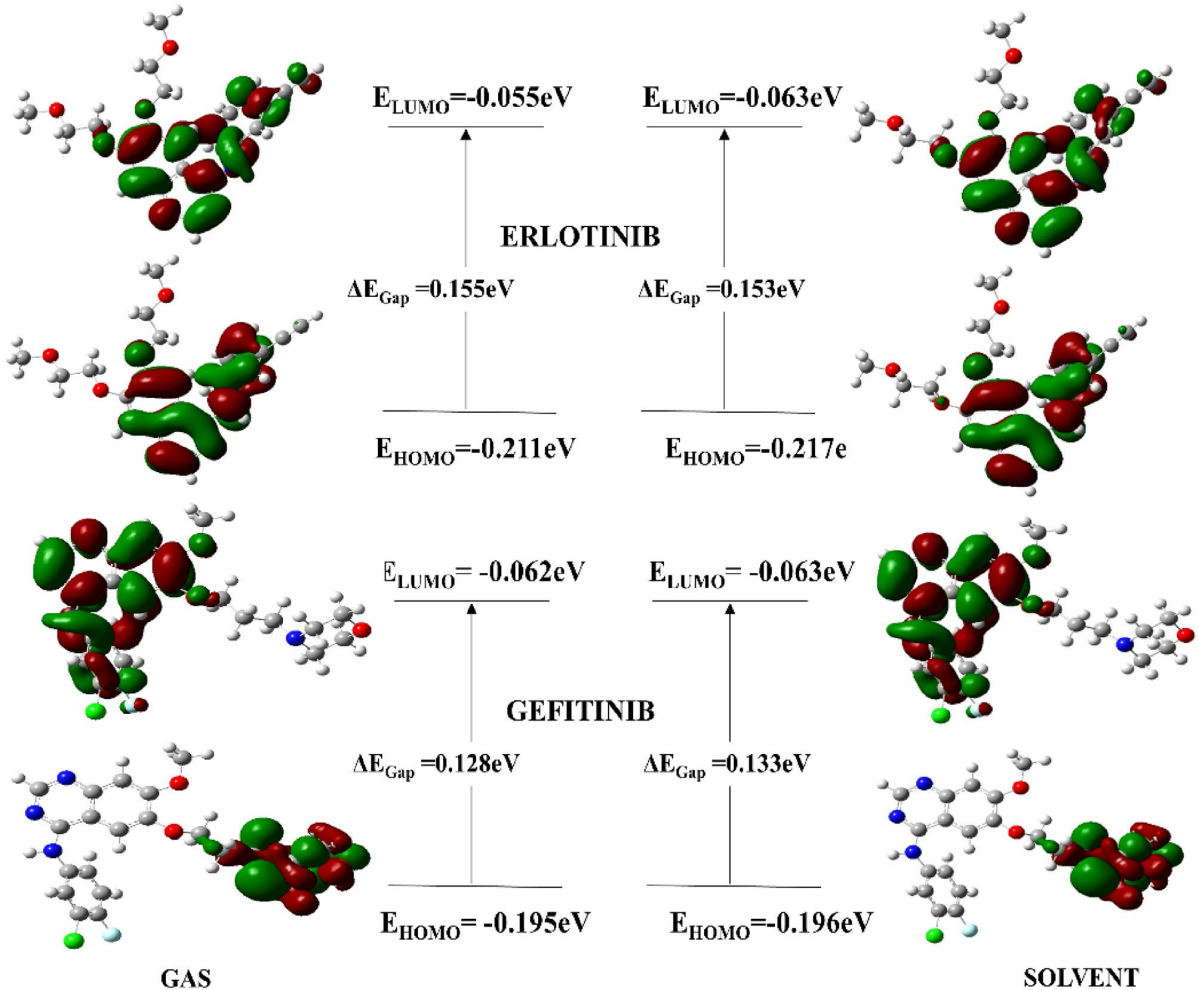
HOMO/LUMO orbitals of Erlotinib and Gefitinib.

The FMOs analysis of Erlotinib and Gefitinib revealed that electron density of HOMO orbitals for Erlotinib was concentrated to quinazoline ring which indicated that reactive character of Erlotinib was due to quinazoline moiety. It was observed that electron density of LUMO orbitals for Erlotinib was majorily confined toward benzene ring and ethyl group substituted on the benzene ring which is responsible for electron accepting character of the compound. The concentration of HOMO/LUMO orbitals along with hydrophobic and hydrophilic interactions contribute toward accumulative chemical reactivity of the compound. In terms of Gefitinib, electron density of HOMO orbitals were confined toward morpholine ring and propoxy group but LUMO orbitals were concentrated toward quinazoline ring of the compound. The HOMO/LUMO orbitals of both drugs are shown in Fig. 5.

### Molecular docking studies

Molecular docking studies provide significant insight into static interactions between compound and targeted protein. Two docking software were used in current study to assess the binding orientation of selected drugs with targeted protein. Both docking programs were assessed for their reliability in predicting the docking scores. It was observed that the AutoDock produced more consistent and better docking scores than MOE. Therefore, the current study further explores the molecular interaction of top ranked poses obtained from AutoDock. The binding affinities of the best pose were also assessed through predicted inhibitory constants (ki) obtained through the AutoDock. The Discovery Studio Visualizer 17.2^24^ and PyMol^25^ was used for visualization of top ranked poses. Only four FDA-approved drug exhibited excellent binding energies and formed a stable protein–ligand conformation. The FDA drugs i.e., Alectinib, Crizotinib, Erlotinib and Gefitinib showed comparatively good docking scores as shown in Table 3. Particularly, Alectinib showed highest docking score of − 32.76 kJ/mol. The docking scores and binding interaction analysis of other drugs is provided in Supplementary Data (Table S1–S3. Figs. S16–S19).

**Table 3:** Docking scores of Alectinib, Crizotinib, Erlotinib and Gefitinib obtained from MOE and AutoDock.

Compound	MOE binding energies (kJ/mol)	AutoDock binding energies (kJ/mol)	Predicted inhibitory constant value (*ki* µM/mM)
Alectinib	− 30.32	− 32.76	2.97
Crizotinib	− 28.53	− 30.54	45.69
Erlotoinib	− 25.22	− 29.70	120.43
Geftinib	− 25.17	− 28.45	122.33
ADP (Co-crystal ligand)	− 16.40	–	3.90 (mM)

The detailed bonding and nonbonding interactions of Alectinib, Crizotinib, Erlotinib and Gefitinib are tabulated in Table 4.

**Table 4:** Hydrogen /hydrophobic interactions observed during molecular docking studies.

Drug	Interaction type	Amino acids	Distance H-A (Å)	Distance D-A (Å)	Angle (º)	Donor atom	Acceptor atom
Alectinib	Hydrogen bonding	LYS63, ARG160, LEU180, ASP179	2.55, 2.79, 2.72, 2.41	3.46, 3.48, 3.32, 3.01	149.29, 125.89, 118.71, 110.21	676[N3+], 2323[Ng+], 4315[N3]	4318[O3], 4308[O2], 2624[O2]
	Hydrophobic interactions	Amino acid residues					
		PRO200, ARG207, TYR213, SER204, MET203, TYR202, ILE109, PHE45, CYS79, ILE83					
Crizotinib	Hydrogen bonding	ASP161	2.23	3.21	160.28	4301[N3]	2344[O2]
	Hydrophobic interactions	Amino acid residues					
		PHE45, LEU180, ILE83, ILE67, MET71, CYS79, LEU180, ARG160, LEU86, LYS63, GLU82					
Erlotinib	Hydrogen bonding	ARG160, ARG207, TYR213	3.38, 2.83, 3.13	4.09, 3.28, 4.01	128.99, 107.49, 147.13	2323 [Ng+], 2786 [Ng+], 2876 [Nam]	4306 [O2], 4301 [O2], 4309 [O3]
	Hydrophobic interactions	Amino acid residues					
		GLU82, ILE83, LEU86, ARG160, TYR202, MET203, PRO200, CYS79					
Gefitinib	Hydrogen bonding	ARG160	3.00	3.30	101.13	2323[Ng +]	4289[N2]
	Hydrophobic interactions	Amino acid residues					
		LEU180, ARG207, TYR213, GLU82, LEU86, ILE83, CYS79, PRO200, MET203					

#### Binding interaction analysis of NEK7-Alectinib complex

**Figure 6:**
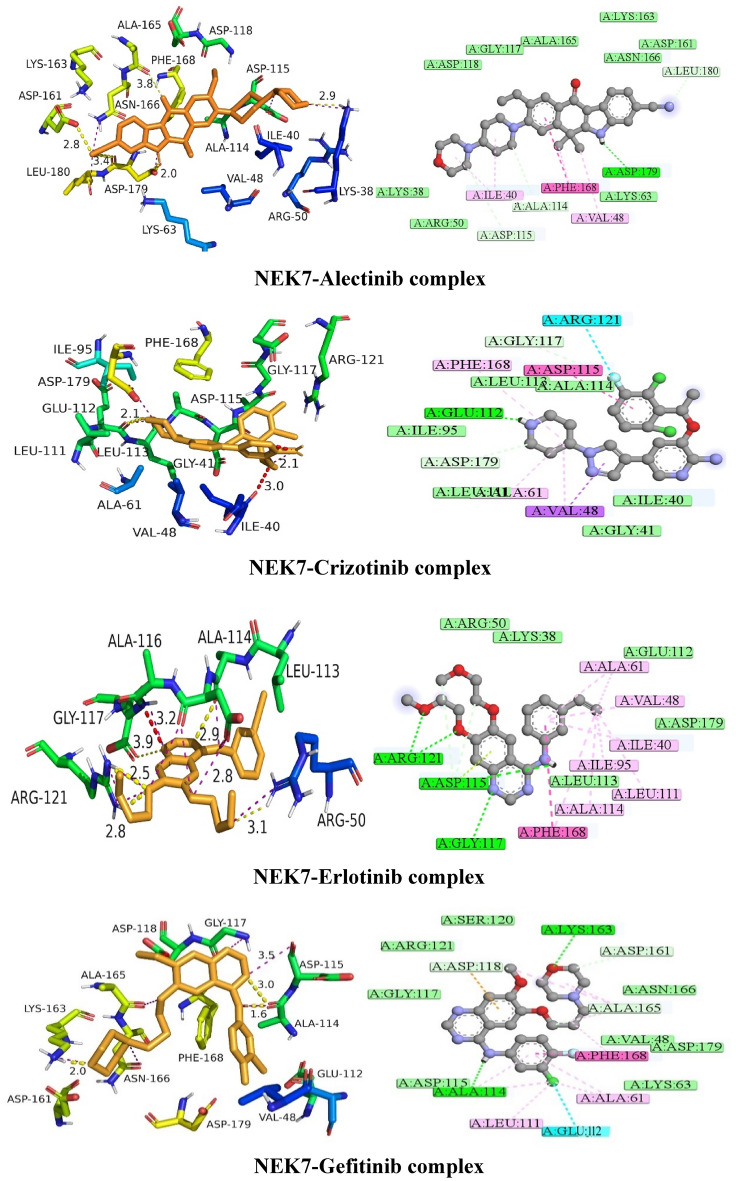
The presumed 3D and 2D binding interactions of selected FDA approved drugs.

The docked conformation of NEK7-Alectinib complex produced substantial molecular interactions with docking score of − 32.76 kJ/mol and a predicted inhibition constant (ki) of 2.97 µM. The odds-on amino acids involved in bonding interactions with Alectinib were as follows; ASP179, LYS63, VAL48, PHE168, ALA114, ASP115, ILE40, ARG50, LYS38, ASP118, GLY117, ALA165, ASN166, LEU180, ASP161, and LYS163. Alectinib exhibited strong molecular interactions, comprising hydrogen bonding, alkyl, pi-alkyl, carbon-hydrogen bonding, and van der Waals interactions. All of these interactions contributed to the stabilization of the protein–ligand complex. In brief, it was discovered that the 4-morpholine ring and the piperidinyl ring of Alectinib were interacting with ILE40 through an pi-alkyl bond. Carbon hydrogen bonds were also formed between the morpholine and piperidinyl rings and ASP115 and ALA114, respectively. In addition, benzocarbazole and the 3-carbonitrile ring generated significant molecular interactions, such as T-shaped, alkyl, conventional hydrogen bonds, and carbon hydrogen bonds with PHE168, VAL48, ASP179, and LEU180, respectively. In addition, van der Waals interactions were observed with GLY117, ASP118, ALA165, LYS163, ASP161, ASN166, LYS63, and ARG50. Figure 6 is showing the putative 2D and 3D binding interactions of Alectinib and other FDA drugs with NEK7. The detailed binding interactions analysis of other FDA drugs against NEK7 and NEK2 are provided in a Supplementary File.

### Virtual screening of compounds library

The most reliable and powerful interactions were discovered between Alectinib and the NEK7 through molecular docking studies conducted on FDA drugs. These results served as the foundation for choosing and obtaining Alectinib structural analogues from the PubChem database. From PubChem, 675 compounds with similarity indices above 80% were retrieved and virtually tested against the NEK7. The virtual screening technique was carried out using Autodock Vina^26^. Only single molecule (146476703) exhibited powerful and persistent interactions, and its docking score of − 34.72 kJ/mol was better to that of Alectinib (detailed interactions are given in Table 5). The identified hit was further subjected to ADMET prediction using MolDesigner, which is an interactive platform for efficient drug development utilizing deep learning algorithms^27^. Moreover, dynamic view of molecular interactions was obtained by MD simulations.

**Table 5:** Molecular interactions observed between 146476703 and NEK7.

Compound	Interaction type	Amino acids	Distance H-A (Å)	Distance D-A (Å)	Angle (°)	Donor atom	Acceptor atom
146476703	Hydrogen bonding	ARG160	2.35	3.03	117.28	632 [O3]	3 [N1]
		ASP78	3.51	4.0	109.66	1486[Ng]	57[O2]
	Hydrophobic interactions	Amino acids residues			Distance (Å)		
		ILE40, VAL48, ALA61, ASP78, LEU111, ASP161, PHE168			3,75, 3.77, 3.66, 3.98, 3.40, 3.96, 3.76		

#### Binding interaction analysis of NEK7-146476703 complex

**Figure 7:**
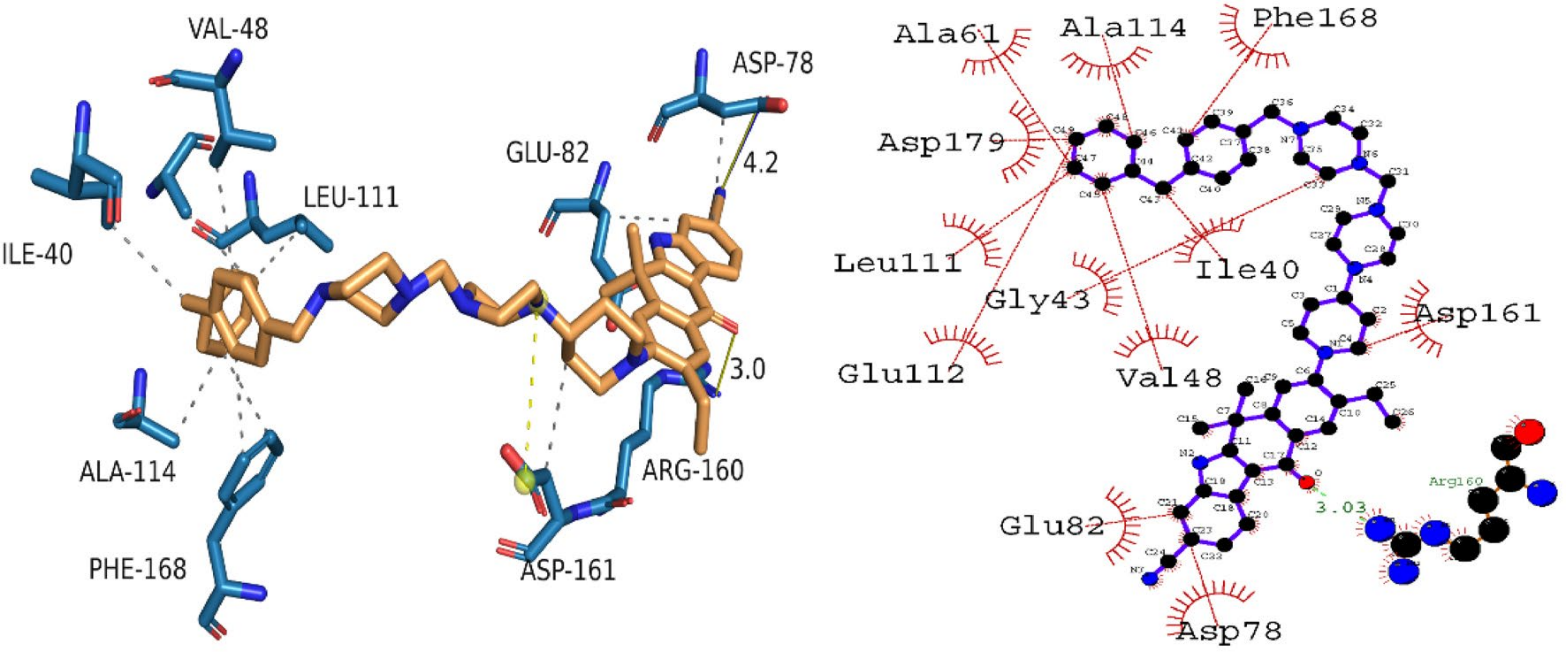
The 3D and 2D binding interactions of compound 146476703 with NEK7.

The docked conformation of the identified hit demonstrated stable intermolecular interactions with the targeted protein. The amino acid residues involved in molecular interactions were as follows; PHE168, ALA114, ALA61, ASP179, LEU111, GLU112, GLY43, VAL48, GLU82, ILE40, ASP161, ASP78 and ARG160. Concisely, two important bonding interactions were implicated in stabilizing the complex. Specifically, the hydrogen bond produced with ARG160 was an important interaction with a bond length of 3.0 Å. In addition, hydrophobic interactions like alkyl, π-alkyl and van der Waals were also stabilizing the complex. Furthermore, single salt bridge interaction was also observed between ligand atom 4 and ASP161 of targeted protein. The binding score of the compound was found to be − 34.72 kJ/mol. The Fig. 7 shows the putative binding mode of compound 146476703 with NEK7.

### Molecular dynamic simulations

The root means square fluctuation (RMSF), root mean square deviation (RMSD) and protein–ligand interactions were calculated through MD trajectory analysis. The Root Mean Square Deviation (RMSD) is a statistic used to calculate the average change in atom displacement in comparison to a reference frame. Examination of RMSD over simulated time provides sufficient insight into structural deviation of protein–ligand complex. RMSD demonstrate the fluctuation of complex with respect to reference frame.

The MD simulation for the NEK7-Alectinib complex was performed in triplicate to validate the docking results. Initially, the main MD simulation run was performed for 200 ns, followed by another two production runs each for 50 ns. Longer MD simulations provided deep insight into the stability of protein–ligand complexes. The average RMSD values obtained from 200 ns, 50 ns and 50 ns production runs were 4.9, 4 and 4.6 Å respectively. The analysis of main production run (NEK7-Alectinib complex) is discussed below and detailed analysis of other two production runs is provided in the supplementary file (Figs. S1, S2). In addition, detailed MD simulation analysis of second best complex NEK7-Crizotinib is also provided in the Supplementary File (Figs. S5–S12).

#### RMSD analysis of NEK7-Alectinib complex

**Figure 8:**
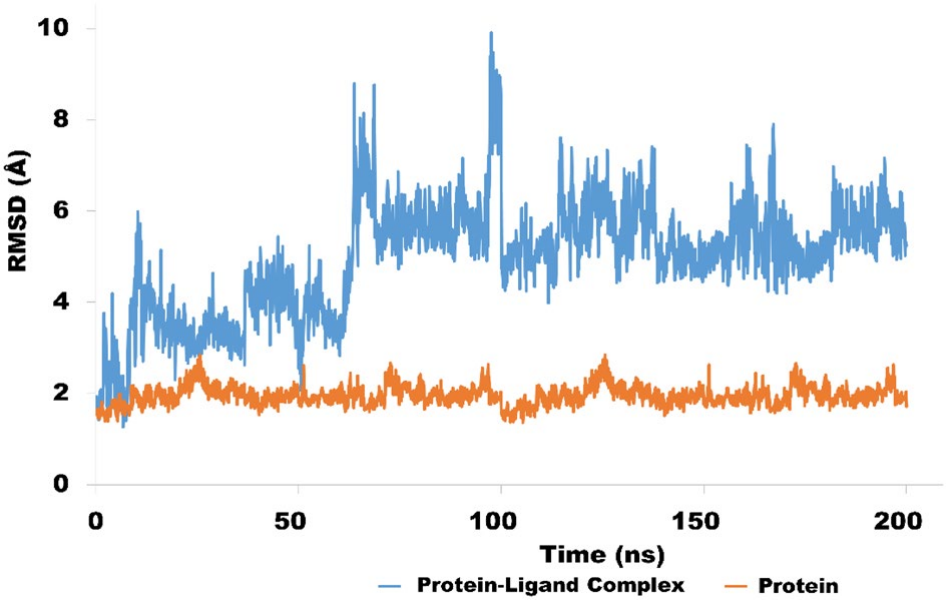
Evolution of RMSD pattern for NEK7 protein (brown colored trajectory) and NEK7-Alectinib complex (blue colored trajectory).

This section display the RMSD value of C alpha atoms of protein with respect to time. The RMSD plot (Fig. 8) is depicting the evolution of RMSD pattern for protein and protein–ligand complex with respect to time. The initial ligand bounded protein complex showed fluctuations around 4 Å for a period of 60 ns. Afterwards, the RMSD pattern showed fluctuation and jumped to 7.8 Å for a period of 10 ns. These fluctuations only last for 10 ns, after which the RMSD pattern gets equilibrated around 5 angstrom for a period of 100 ns. It was observed that after 100 ns of simulation time, the RMSD pattern was slightly stable as compared to the first half of the simulated trajectory. After 100 ns, significant contacts were produced especially with ASP118, ALA165, ASN166 and LEU180 which stabilized the complex. The average RMSD for protein ligand complex was 4.9 Å, and fluctuations were also within acceptable limits. In terms of protein RMSD pattern, the NEK7 protein showed a much better RMSD pattern and remained stable throughout the simulated trajectory. The average RMSD value for protein was 1.9 Å, which is quite acceptable. These findings provide insight into the molecular interactions of protein–ligand complexes. Concisely, relatively stronger interactions were observed in the second half of production runs. Similarly, the second and third production runs (Supplementary File) also showed fewer fluctuations. These findings provide testimony that the ligand remained sufficiently bound to the receptor throughout the simulated time.

#### RMSD analysis of NEK7-146476703 complex

**Figure 9:**
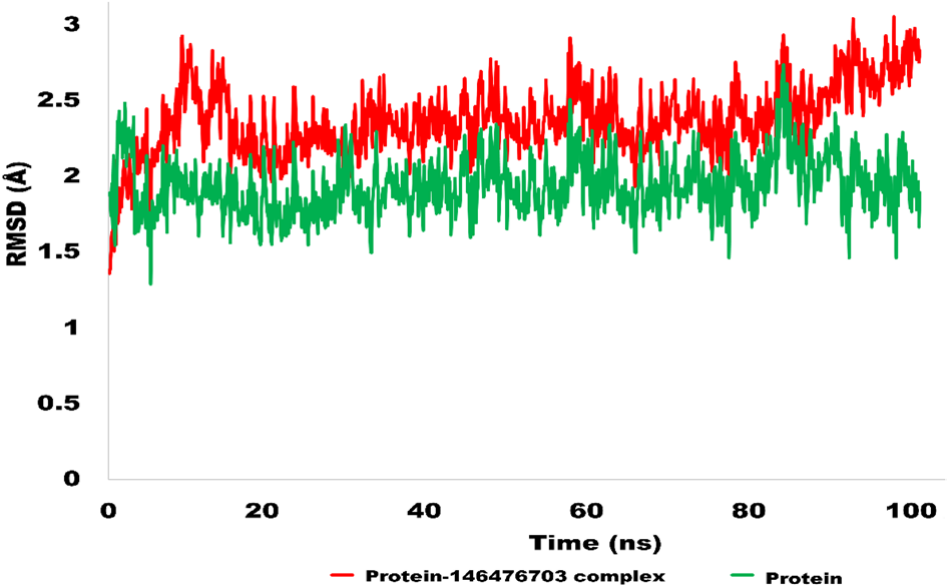
Evolution of RMSD pattern for protein and protein-146476703 complex.

The stability of protein-146476703 complex was investigated via MD simulation studies. The Desmond software was utilized for the production run of 100 ns. The resulting RMSD trajectory was inspected for determining the structural deviations and nature of molecular interactions. The evolution of RMSD pattern indicates the protein–ligand complex was equilibrated well as the fluctuations remained below 3 Å. The average RMSD value was calculated to be 2.9 Å which is considered as stable and deemed to be equilibrated. The Fig. 9 is showing the evolution of RMSD pattern for protein–ligand complex as a function of time. Therefore, it is deduced that hit molecule produced stronger contacts with amino acid residues of active site and the docking results are quite validated by MD simulations.

#### Root mean square fluctuation (RMSF) analysis of NEK7

**Figure 10:**
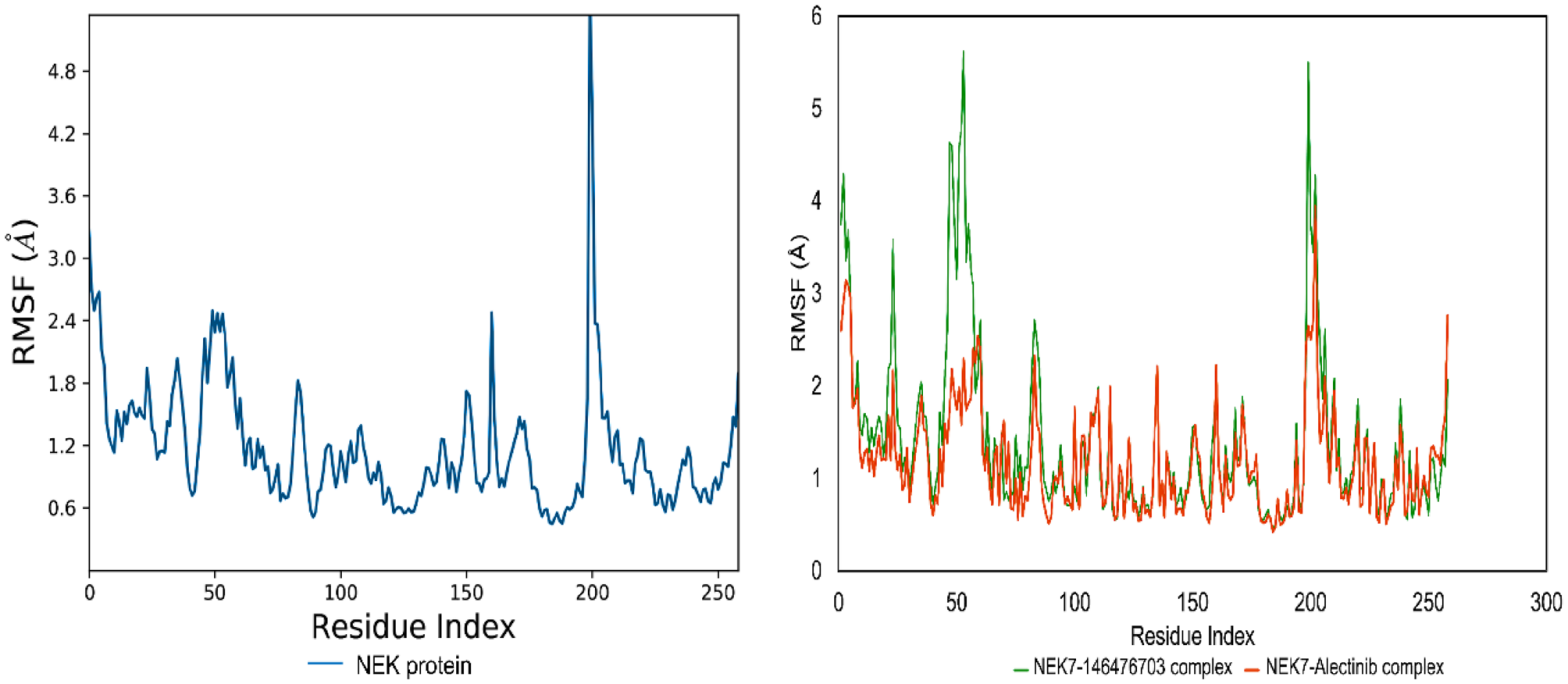
Root mean square fluctuations (RMSF) of NEK7 protein (left) and NEK7 bound to Alectinib and 146476703 (right).

The RMSF is helpful for characterizing local modifications in the protein chain. Low RMSF values of binding site residues demonstrate the stability of ligand binding to the protein. Figure 10 depicts the RMSF of NEK7 amino acid residues in the presence and absence of Alectinib and 146476703. In general, the NEK7 residues remained below 2 Å in both states, demonstrating their stability. Nevertheless, some protein residues exhibit large changes, notably amino acid residues in the range of 180 to 220. These residues are located in the protein’s C-terminal region. Alpha helices and beta strands are often more rigid than the unstructured portion of a protein and more stable than loop regions. Regarding the NEK7-Alectinb complex, Alectinib-bound NEK7 residues exhibited a steady pattern, and the RMSF stayed below 2 Å. Similarly, the NEK7-146476703 complex maintained a steady pattern throughout the trajectory simulation and did not reach 2.5 angstroms. The significant fluctuations of RMSF values of Cα atoms for amino acid residues numbered from 40 to 80 were observed for the side residues chains of amino acids not bound to the ligand. However, the amino acids bound to the ligand did not show significant atomic fluctuations during the MD simulation.

**Figure 11:**
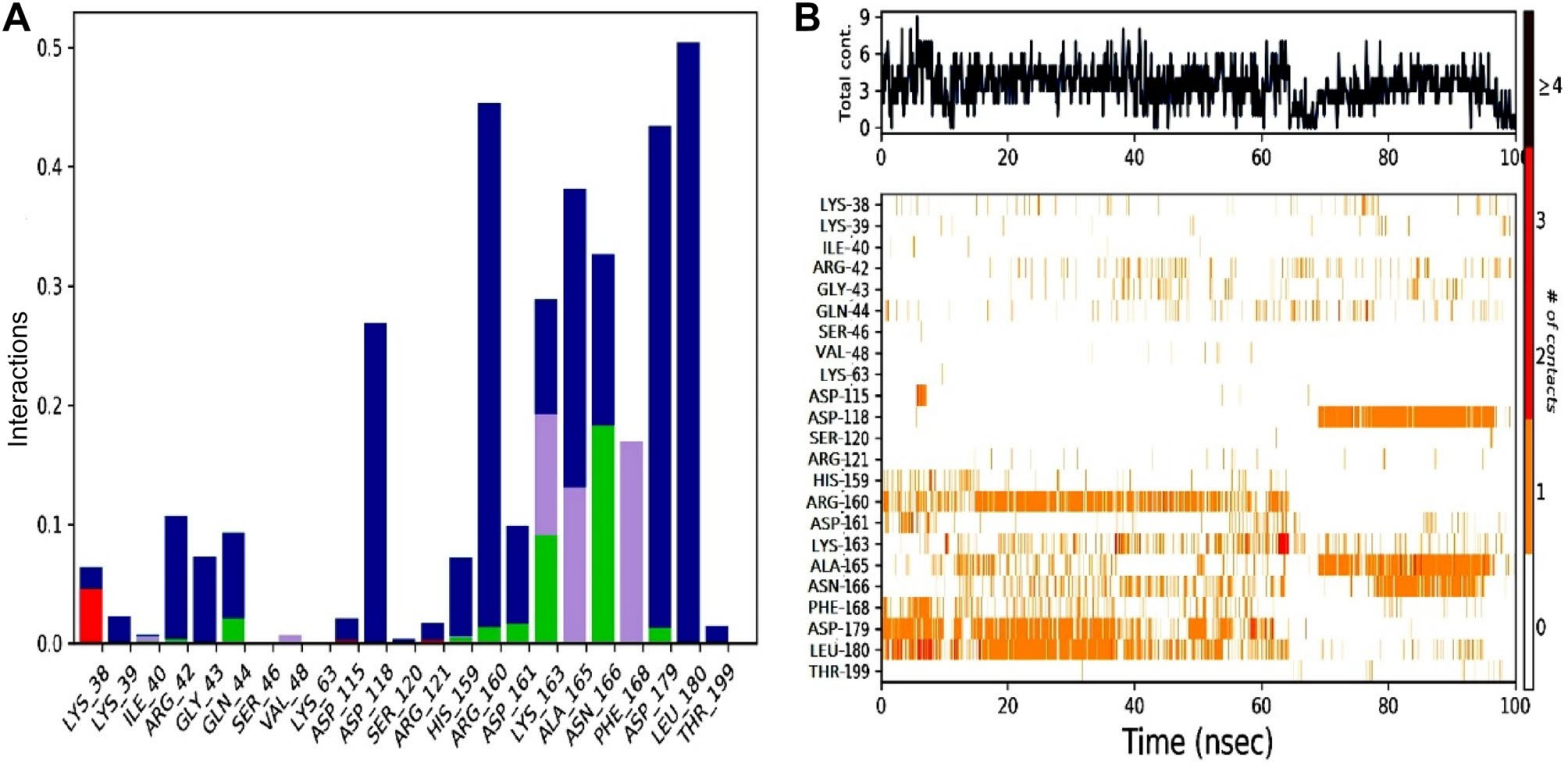
(**A**) Protein–ligand (NEK7-Alectinib) contact histogram (H-bonds, Hydrophobic, Ionic, Water bridges), extracted from respective 100 ns MD trajectories. The stacked bar charts are standardized and normalized along the trajectory; for example, a value of 0.8 implies that a certain interaction is maintained for 80% of the simulation time. (**B**) Representation of interactions and contacts of H-bonds plotted against time. The graph illustrates the SSE composition for each trajectory frame during the simulation (provided in Supplementary File Figs. S3, S4). The top panel gives the overall contact between protein and ligand. Some of this ligand can have more than one contact (Dark orange) (Desmond software^40^).

During the course of simulation time, different types of ligand interactions were observed with targeted protein. This interaction included hydrogen bonding, hydrophobic, ionic bonds and water bridges. Figure 11 showing the different types of interactions. It can be observed that amino acid residues of NEK7 protein was involved in multiple hydrogen bonds and different types of hydrophobic interactions were also involved in stabilizing protein–ligand complex. In case of NEK7-Alaectinib complex, ASN166 and LYS163 were important residues in terms of hydrogen bonding. Moreover, amino acid PHE168 was engaged through hydrophobic interaction for 30% of simulation time. Whereas, LEU180 was involved in water bridges for 90% of simulated trajectory. In addition, for stacked bar chart demonstrating the time of contact maintained over the course of simulation time. A value of 1 showed that contact is maintained for 100% simulation time. Values above 1.0 are feasible.

**Figure 12:**
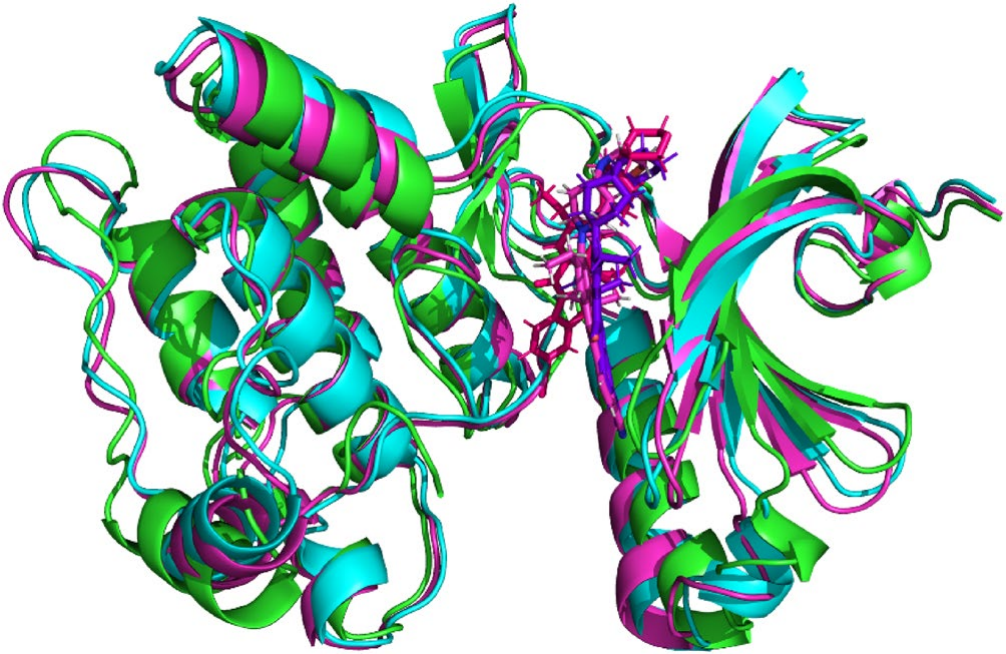
Aligned structures of NEK7-Alectinib during simulations; Green protein and hot pink ligand at 0 ns; blue protein and blue ligand at 90 ns; pink protein and light pink ligand at 200 ns (PyMOL^25^).

The trajectory analysis of Alectinib-NEK7 complex revealed that ligand remained attached to active pocket and ddidnt deviate from activation loop. The super aligned structures of liganded protein obtained at different intervals is illustrated in Fig. 12.

#### MMGBSA energies of simulated trajectories

The molecular mechanics Poisson-Boltzmann surface area (MM/PBSA) and molecular mechanics generalized born surface area (MM/GBSA) are promising methods for determination of free Binding energies during MD simulations. Since they are modular and do not require training set calculations. They have been used effectively to reproduce and justify experimental data, as well as to enhance the outcomes of virtual screening and docking. But they have a number of wrong and unlikely calculations, such as the lack of information on conformational entropy and the number and free energy of water molecules at the binding site^28,29^.

$$\Delta {\text{Gbind }} = \, \Delta {\text{E mm }} + \, \Delta {\text{G sol }} + \, \Delta {\text{G SA}}{.}$$

The MMGBSA energies for all the four complexes were determined through Thermal_mmgbsa script of Schrodinger. MMGBSA Energies are tabulated in Table 6.

**Table 6:** MM-GBSA binding energies of Alectinib, Crizotinib, Gefitinib and Erlotinib.

Drugs	ΔG_bind_ (kJ/mol)	ΔE_coulomb_ (kJ/mol)	ΔE_covalent_ (kJ/mol)	ΔE_H-bond_ (kJ/mol)	ΔE_vdW_ (kJ/mol)	Lipophilic energy (kJ/mol)	Sol_GB (kJ/mol)
Alectinib	− 303.68	283.29	0.67	− 8.61	− 117.77	− 52.55	− 242.12
Crizotinib	− 274.09	− 8.82	2.53	− 4.000	− 134.81	− 138.57	21.63
Gefitinib	− 266.21	− 48.51	8.41	− 1.74	− 186.11	− 110.45	91.04
Erlotinib	− 260.17	15.65	14.31	− 0.61	− 167.06	− 146.77	26.21

#### SeeSAR analysis

**Figure 13:**
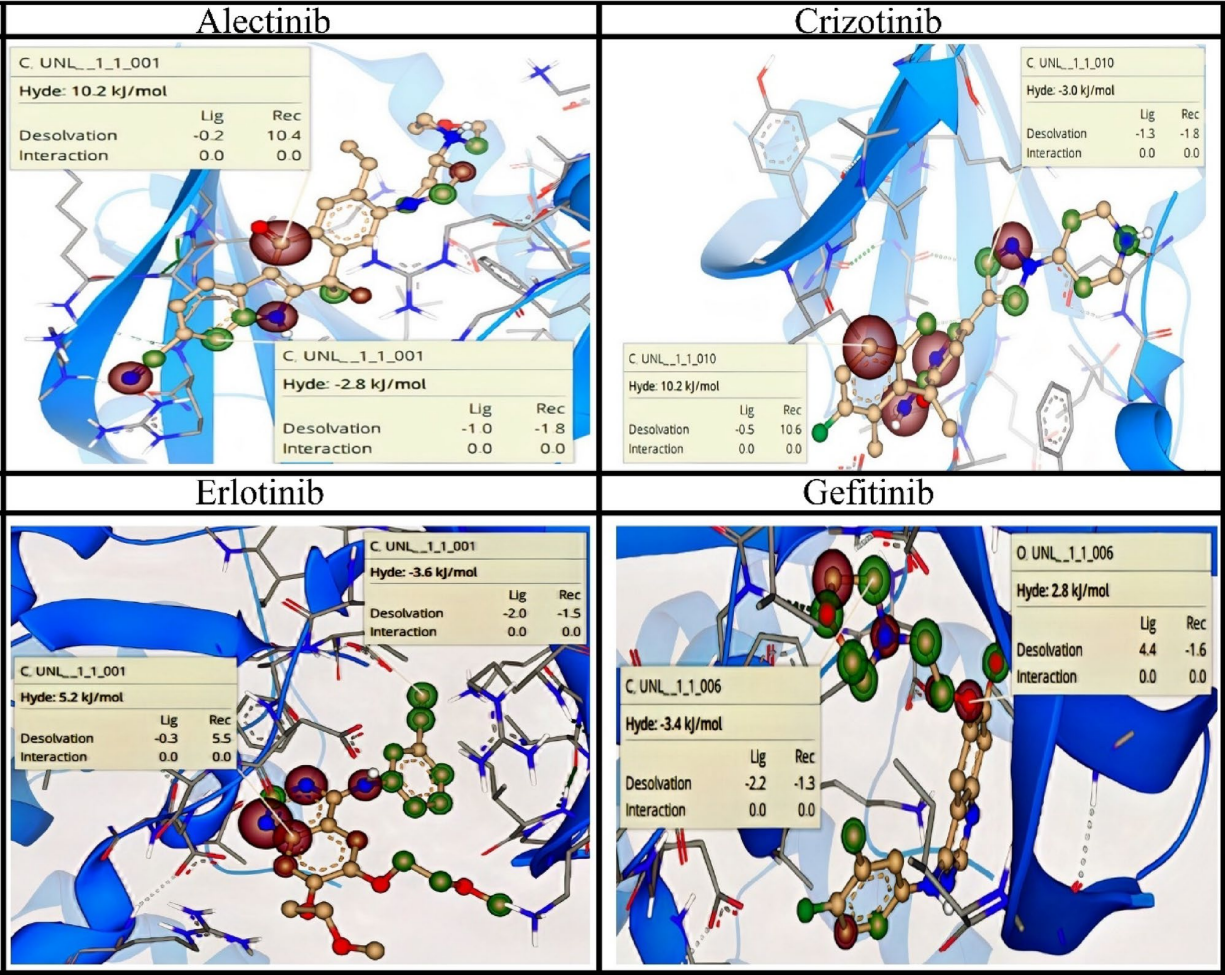
SeeSAR analysis of top ranked FDA drugs (seeSAR 12.0.1^46^).

The visual depiction of binding affinities of docked conformations was illustrated through SeeSAR analysis. SeeSAR analysis was performed using SeeSAR version 12.0.1. Coronas represented in green and red colors were used to depict the structural features of compounds contributing toward binding affinity. Structural characteristics that improve binding affinity are represented as green colored coronas, while those features that reduce binding affinity are represented as red colored coronas. Structural component of the compounds having no contribution toward binding affinity remained colorless. It was observed that benzene ring was contributing favorably toward binding affinity of Alectinib with Hydrogen bond and Dehydration (HYDE) energy of − 2.8 kJ/mol. Similarly halogen substituted benzene ring was also contributing toward binding affinity of compound. SeeSAR analysis of FDA approved drugs is shown in Fig. 13.

#### Normal mode analysis (NMA) of apo protein and liganded protein

NMA (Normal Mode Analysis) is a computational technique used to study the conformational changes in proteins. The NMA analysis performed on the apo protein and protein-alectinib complex, as well as the Nek7-compound 146476703 complex via IMODS webserver^30^. The findings of NMA investigations revealed some notable differences in the deformability of these systems. The main-chain deformability is a property that describes the flexibility of a molecule, specifically the ability of its main chain to change its shape at each residue (amino acid) along the chain. The main chain refers to the backbone of a protein molecule, which is composed of repeating units of nitrogen and carbon atoms. The deformability of a molecule is an important factor that determines its function and stability. The apo NEK7 protein was found to be highly deformable with a deformability score of 0.9. This is indicated by the presence of large peaks in the deformability profile, suggesting that the protein is able to undergo significant conformational changes at these sites. This high deformability of the apo protein is likely due to the lack of any stabilizing interactions with other molecules, such as ligands or inhibitors. In the absence of these interactions, the protein is free to adopt a wide range of conformations, resulting in its high deformability.

**Figure 14:**
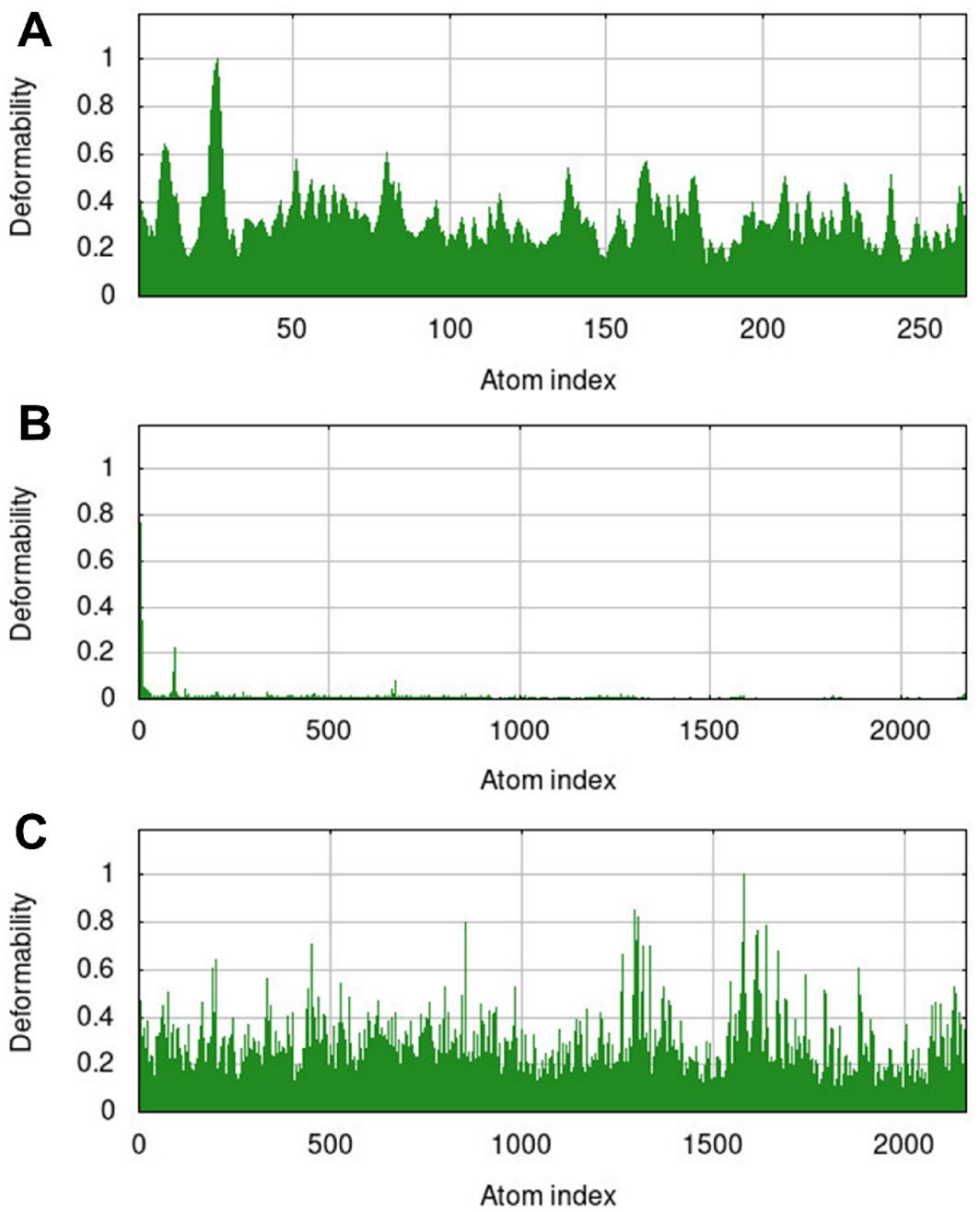
Deformability potential of apo protein (**A**), the NEK7-alectinib complex (**B**), and the NEK7-compound 146476703 complex (**C**).

The protein-alectinib complex was found to be stiff and showed no significant peaks of deformability (Fig. 14). This suggests that the presence of the alectinib molecule has significantly impacted the conformational freedom of the protein, leading to a reduction in its deformability. This is expected, as the alectinib molecule is likely to form stabilizing interactions with the protein that restrict its ability to change shape. The Nek7-compound 146476703 complex was found to have a deformability score of 0.6, which is less than that of the apo protein. This indicates that the presence of the compound 146476703 has also had an impact on the deformability of the protein, although to a lesser extent than alectinib. This reduction in deformability is likely due to the formation of stabilizing interactions between the compound and the protein, similar to those observed for the protein-alectinib complex. conclusively, the NMA analysis performed on the apo protein and protein-alectinib complex, as well as the Nek7-compound 146476703 complex, provides valuable insights into the conformational changes of these systems. The results indicate that the presence of both alectinib and compound 146476703 has an impact on the deformability of the protein, resulting in more rigid and stiff structures. These findings are important for understanding the molecular mechanisms underlying the interactions between the protein and these molecules, and could have implications for the development of new therapeutic strategies for the treatment of diseases.

#### Covariance plots

Covariance plots are a useful tool for visualizing the structural interactions between different residues in a protein. In this study, covariance plots were calculated for the apo NEK7 protein, the NEK7-alectinib complex, and the NEK7-compound 146476703 complex. The covariance plot of the apo NEK7 protein showed a mix of red, blue, and white regions. The red regions indicate high positive covariance, meaning that the residues in these regions tend to move together in a coordinated manner. On the other hand, the blue regions indicate high negative covariance, meaning that the residues in these regions tend to move in opposite directions. The white regions indicate low covariance, meaning that the residues in these regions do not tend to move together or in opposite directions.

The covariance plot of the NEK7-alectinib complex^30^ showed the highest concentration of red regions, indicating high levels of positive covariance between residues in this complex. This suggests that the alectinib molecule is forming strong interactions with the protein, leading to coordinated movements of residues in specific regions. The blue regions were also significant, indicating that the formation of these interactions may also lead to the restriction of certain movements in other regions of the protein.

**Figure 15:**
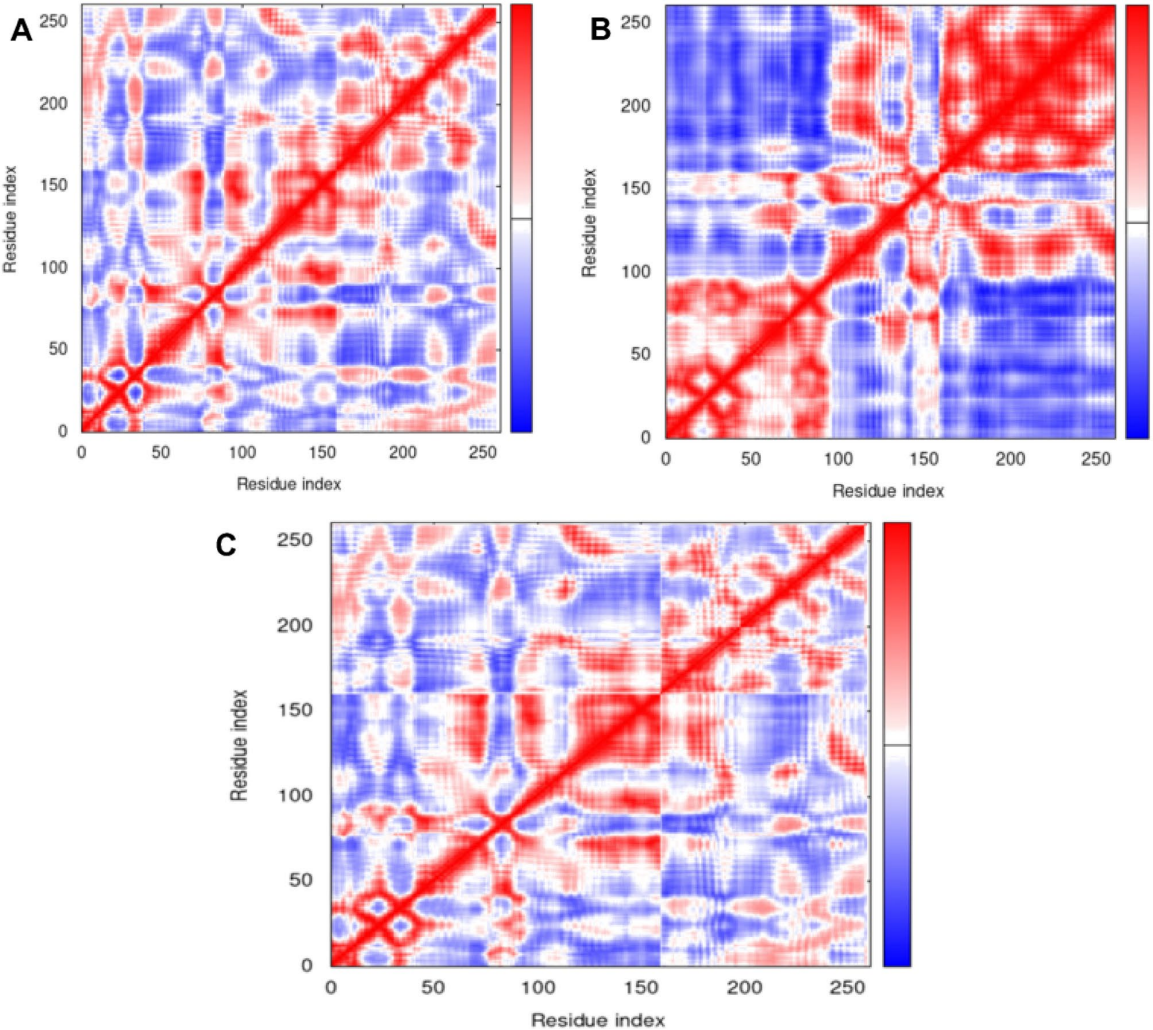
Covariance plot for the apo NEK7 protein (**A**), the NEK7-alectinib complex (**B**), and the NEK7-compound 146476703 complex (**C**) (IMODS^30^).

The covariance plot of the NEK7-compound 146476703 complex showed a similar pattern to that of the NEK7-alectinib complex, with high levels of positive covariance indicated by the red regions (Fig. 15). However, this plot had a slightly lower level of interactions compared to the NEK7-alectinib complex, as indicated by the lower concentration of red regions. The covariance plots provide valuable insights into the structural interactions between residues in the apo protein, the NEK7-alectinib complex, and the NEK7-compound 146476703 complex. The results suggest that the presence of both alectinib and compound 146476703 leads to the formation of significant interactions with the protein, leading to coordinated movements of residues in specific regions and the restriction of movements in others. The detailed NMA analysis is rovided in Suplementary File (Figs. S13–S15).

### ADMET properties

Virtual screening of the 675 compounds library identified compound 146476703 as a potential hit. It is inevitable to predict the in-silico ADMET properties of an identified hit. So the current study has utilized MolDesigner^27^ for prediction of the pharmacokinetic profile of the compound. MolDesigner is an interactive tool for the production of efficacious drugs with a deep learning model, i.e., MPNN. The solubility and lipophilicity of compound 146476703 were predicted to be − 4.36 log mol/L and 3.37 (log-ratio) respectively. The detailed ADMET profile is tabulated below (Table 7).

**Table 7:** Predicted ADMET property of compound 146476703.

Property	Predicted value
Solubility	− 4.36 log mol/L
Lipophilicity	3.37 (log-ratio)
(Absorption) HIA	98.12%
(Absorption) Pgp	83.52%
(Absorption) bioavailability F20	77.57%
(Distribution) BBB	85.85%
(Distribution) PPBR	73.40%
(Metabolism) CYP2C19	65.72%
(Metabolism) CYP2D6	83.32%
(Metabolism) CYP3A4	43.87%
(Absorption) Caco-2	− 5.27 cm/s
(Metabolism) CYP1A2	14.61%
(Metabolism) CYP2C9	20.85%
(Excretion) half life	7.93 h
(Excretion) clearance	8.19 mL/min/kg

## Methodology

### Density functional theory (DFT) calculations

The Gaussian 09W programme^21^ was used to perform optimization and frequency calculation for selected drugs. DFT calculations were carried out using a linux-based workstation equipped with an AMD Ryzen 9 processor running @ 3.64 GHz and 64 GB of RAM memory. The structural geometries of selected drugs were optimized using DFT/B3LYP functional correlation and Karlsruhe-type basis sets (SVP) for appropriate assumptions on drugs’ electronic characteristics and attributes. The B3LYP functional is a hybrid approach that combines the Slater exchange functional, Becke gradient correction, and LYP correlation. It provides a good balance of accuracy and computational efficiency for a wide range of molecular systems. It is widely used due to its ease of implementation and good results for molecular geometries, exchange–correlation energy, and is faster than most post-Hartree–Fock techniques. B3LYP is a robust DFT method with 3 parameters compared to other hybrid functionals with up to 26. In addition, the split valance polarized (SVP) set is a [3s2p] contraction of a [7s4p] set of primitive functions reliable for attianig the stable configuration of compounds. The SVP basis set promisingly covers 1st and 2nd row elements of periodic table however the coverage of elements depend upon level of contraction applied to a basis set. Furthermore, using the same level of theory, the frontier molecular orbital (FMO) analysis, reactivity descriptors and chemical reactivity was also determined. Gauss View 6^31^ was used to analyze the output files.

### Molecular docking studies

Molecular docking is an effective approach for determining the binding orientation and binding affinities of compounds against targeted protein. Molecular docking studies were performed using Molecular Operating Environment (MOE) 2015.10^32^ and AutoDock 4.2^33^. The present study has employed two docking software in order to improve the accuracy and reliability of docking results. A crystallographic structure of NEK7 (PDB ID = 2wqn; resolution = 2.30 Å) was retrieved from RCSB protein data bank (https://www.rcsb.org/)^34^. The 2D structures of all FDA approved drugs were generated using IUPAC names obtained from PubChem (https://pubchem.ncbi.nlm.nih.gov/). In addition, 675 compounds library were also retrieved form PubChem database and subjected to virtual screening against NEK7 utilizing the proposed approach. All compounds were subjected to preliminary energy minimization process in order to remove any steric clashes. The prepared compounds library was saved to required database for docking against targeted protein^35^. Protein preparation was carried out using MOE protein preparation utility. Protein preparation included removal of het and water molecules. Missing residues were repaired by 3D protonating the protein structure and correcting the identified issues at pH 7 and 300 K temperature. Furthermore, polar hydrogens were added with standard 3D geometry and gasteiger partial charges were assigned using MMFF94x forcefield^36^. Afterwards, the active site residues were identified using the dimensions of co-crystal ligand (ADP) utility and dummies atoms were generated at respective alpha spheres. Selected amino acid residues of active site were as follows; LYS63, ARG160, LEU180, ASP179, LEU113, ILE40, GLY41, GLY117, ALA61, ALA116, ALA165, ASP118, GLY43, LEU111, PHE168, ASP115, ASN166, SER46, GLU82 ARG121, GLY112, ALA114, PRO200, ARG207, TYR213, SER204, MET203, TYR202, ILE109, PHE45, CYS79, ILE83 and VAL48. Finally, docking was performed using the MOE default parameters, with a triangular matcher as placement method and refinement was set to induce fit. The scoring function was set to London dG and total 100 poses were generated for each compound^37^. The docking protocol was validated by re-docking co-crystal ligand, and the conformation with RMSD value less than 2 angstrom was selected for further analysis^38,39^.

Protein preparation process was also undertaken in AutoDock including addition of polar hydrogen, removal of water and hetero atoms. A grid box centered at dimensions (X; -11.348222, Y; -32.512407, Z; -46.605296) of co-crystal ligand (ADP) was generated and molecular docking was performed using Lamarckian genetic algorithm^37^. Both software’s were accessed for reliability of docking scores. The docking output from both software’s are tabulated in Table 3.

### Molecular dynamics simulation

Schrodinger LLC’s Desmond^40^ package was used to simulate protein–ligand complexes over 100 ns. Molecular docking facilitates the generation of initial protein–ligand complexes in a static state^41^, whereas MD simulations gravitate to figure out atoms evolutions over time. MD simulations revealed about the orientation of ligand binding in a physiological environment^42,43^.

Maestro protein preparation wizard was utilized for optimization of initial protein–ligand complex. The system was developed using a system builder tool. The system was submerged in a water model (TIP3P) containing an orthorhombic box. For production run, the OPLS 2005 force field was employed^44^. The system was neutralized by the addition of NaCl ions at a concentration of 0.15 M. The system was equilibrated for 1 ns in the NVT ensemble at 300 K temperature and 1 atmospheric pressure. The NPT ensemble was then equilibrated for additional 1 ns. Temperature and pressure were maintained using a Martyna-Tobias-Klein and Noose-Hoover thermostat throughout simulation. The production run was performed for 200 ns^45^.

### SeeSAR analysis

SeeSAR analysis was used to assess the persuasive rationale for binding affinities and Hydrogen bond and Dehydration energies (HYDE) of FDA drugs with NEK7^46^. In SeeSAR software 12.0.1^47^, the top-ranked conformation acquired from molecular docking was subjected to pose generation. Each protein–ligand complex was submitted to the SeeSAR docking mode, which produced many orientations based on binding affinity. Green colored coronas represent structural properties of drugs that contribute positively to binding affinity, whereas red colored coronas represent structural aspects that contribute adversely. The structural properties of the drugs that have no effect on their binding affinity were left colorless.

## Conclusion

The present work has utilized a comprehensive in-silico approach for the repurposing of FDA drugs against the NEK7 protein. The selected drugs, including Alectinib, Crizotinib, Erlotinib, and Gefitinib, were optimized and in-depth electronic and reactivity parameters were estimated using DFT studies. All drugs exhibited reactive potential. Moreover, molecular docking studies revealed the formation of stable protein ligand complexes with all drugs, but Alectinib and Crizotinib produced relatively stronger molecular interactions and produced stable protein–ligand complexes. Moreover, the top-ranked conformation of Alectinib and Crizotinib was subjected to molecular dynamic simulations, which further supported the stability of the protein–ligand complex under experimental conditions. Furthermore, 675 structural analogs of Alectinib were subjected to virtual screening against NEK7, resulting in the identification of compound 146476703 as having potential drug-like properties. It is recommended to further investigate compound 146476703 at the molecular level, with the goal of synthesizing and developing an effective therapeutic approach for NEK7-related cancers and associated malignancies.

## Supplementary Information

Supplementary Information.

## Data Availability

The datasets analysed during the current study are available in the PubChem database and Protein Data Bank repository, (https://pubchem.ncbi.nlm.nih.gov/#query=CID49806720%20structure&tab=similarity; https://www.rcsb.org/structure/2WQN).
