# Supplementary material for: Revisiting the inhibitory potential of protein kinase inhibitors against NEK7 protein via comprehensive computational investigations
Source: Sci Rep. 2023 Mar 15;13:4304. doi: 10.1038/s41598-023-31499-7 (PMC10017757; doi:10.1038/s41598-023-31499-7)
Supplement: Supplementary file 1 — Supplementary Information. [file 41598_2023_31499_MOESM1_ESM.doc]

Revisiting the activities of protein kinase inhibitors as a potential inhibitor of NEK7 via molecular docking approach, DFT studies, and molecular dynamics simulations

Syeda Abida Ejaz^1*^, Mubashir Aziz^1^, Zeenat Zafar^2^,Naveed Akhtar^3^, Hanan A. Ogaly^4,5^

*^1^Department of Pharmaceutical Chemistry, Faculty of Pharmacy, The Islamia University of*

*Bahawalpur, Bahawalpur, Pakistan, 63100*

*^2^Nistar Medical University Multan, Punjab, Pakistan,*

*^3^Department of Pharmaceutics, Faculty of Pharmacy, The Islamia University of Bahawalpur,*

*Bahawalpur, Pakistan, 63100.*

*^4^ Chemistry Department, College of Science, King Khalid University, Abha 61421, Saudi Arabia.*

*^5^ Biochemistry and Molecular Biology Department, Faculty of Veterinary Medicine, Cairo University, Giza 12211, Egypt*

***Correspondence:** abida.ejaz@iub.edu.pk; abidaejaz2010@gmail.com; Tel: +92-062-9250245 Fax: +92-062-9250245

**Results and Discussions**

**Molecular docking interactions of FDA drugs with NEK7**

**NEK7-Crizotinib complex**

The bonding and non-bonding interactions of drug Crizotinib involved important amino acid residues of activation loop of NEK7. The amino acid residues which were involved in molecular interactions with Crizotinib were as follows; ARG121, GLY117, PHE168, ASP115, LEU113, ALA114, GLU112, ILE95, ASP179, LEU111, ALA61, VAL48, GLY41, and ILE40. Crizotinib showed important molecular interactions which contributed to binding affinity and binding score of protein-ligand complex. Most important interactions were observed between piperidinyl, pyrazol and 2,4 dichloro-4-floro phenyl rings of Crizotinib. Piperidinyl ring was involved in major stabilizing interaction i.e., conventional hydrogen bonding with GLU112 of activation loop. Moreover, Piperidinyl ring was also involved in formation of carbon hydrogen bonding, alkyl and π-alkyl interactions with ASP179, ALA61, VAL48 and PHE168 respectively. These interactions were majorly contributing toward increasing the binding energy. Meanwhile, Pyrazole ring was involved in π-sigma bonding with VAL48 amino acid residue. Furthermore, 2,4 dichloro-4-floro phenyl ring was reactive toward amino acid residues of activation loop as it bear halogen atoms which was imparting positive mesomeric effect (***+M***) which was increasing the resonance and rendering the phenyl ring more reactive. It was observed that halogen atom of phenyl ring was interacting with ARG121 and forming carbon hydrogen bond with GLY117. Whereas, resonating electronic cloud of phenyl ring was involved in π stacked interaction with ASP115. Crizotinib showed binding energy of -30.54 kJ/mol. The binding affinity was also good as complex showed predicted inhibitory constant value of (***ki***) 45.69µM. Van der walls interaction are important hydrophobic interactions which were observed with following amino acid residues; ILE95, GLY41, ILE40, ALA113, ALA114 and LEU111. The 2D and 3D interactions of Crizotinib is shown in Figure 6.

**NEK7-Erlotinib complex**

The docked conformation of drug Erlotinib with in active pocket of NEK7 formed stable protein-ligand complex with least binding energy OF -29.70 kJ/mol and ***ki*** 120.43 µM. The amino acid residues which were involved in bonding and nonbonding interactions with Erlotinib were as follows; ARG121, ASP115, GLY117, ASP118, PHE168, ALA114, LEU113, LEU111, ILE95, ILE40, ASP179, VAL48, ALA61, GLU112, LYS68, ARG50. Erlotinib showed important molecular interactions including hydrogen bonding, carbon hydrogen bonding, alkyl, pi-alkyl and van der waal interactions. All these interactions were involved in stabilizing protein-ligand complex. Briefly, it was observed that dimethoxyethane of Erlotinib was forming conventional hydrogen bond with ARG121. It also made carbon hydrogen bond with ASP115. The anisole ring has a pi-lone pair interaction with ASP115. Moreover, the 4, 5-dihydropyrimidine ring was involved in making conventional hydrogen bond with GLY117. Methylbenzene was involved in forming pi-pi stacked interaction with PHE168 and its triple bond was involved in making alkyl interactions with ALA61, VAL48, ILE40, ILE95, ALA114 and LEU111. Furthermore, among non-bonding interactions van der Waals interactions were observed with following residues; ASP118, LEU113, ASP179, GLU112, LYS38, ARG50. These interactions are inferior as compared to Alectinib and Crizotinib

**NEK7-Gefitinib complex**

The bonding and non-bonding interactions of drug Gefitinib involved important amino acid residues of activation loop of NEK7. The amino acid residues which were involved in molecular interactions with Gefitinib were as follows; SER120, LYS163, ASP161, ASN166, ALA165, VAL48, ASP179, PHE168, LYS63, ALA1 61, LEU113, GLU112, LEU111, ALA114, ASP115, GLY117, ARG121 and ASP118. Gefitinib showed important molecular interactions which contributed to binding affinity and binding score of protein-ligand complex. Most important interactions were observed with methoxymethane which showed alkyl interaction with ALA165. Xylene ring was forming pi-anion interaction with ASP118 and divinylamine was making hydrogen bond with ALA114. 1-choloro-2-fluorobenzene ring showed pi-pi stacked interaction with PHE168, the halogen atom showed interaction with GLU112, and pi-alkyl interaction with ALA6 and LEU111. Van der walls interaction are important hydrophobic interactions which were observed with following amino acid residues; SER120, ASN166, VAL48, LYS63, LEU113, ASP115, GLY117, ARG121. The docking score was observed to be -28.45 kJ/mol which is better than Erlotinib but less than Alectinib. So it was concluded that Alectinib was most potent FDA approved drug against NEK7 and producing stable protein-ligand complex.

**Table S1**. Molecular docking scores of FDA approved inhibitors against NEK7 with Predicted inhibitory constant

| **Compound** | **MOE docking score (kJ/mol)** | **Autodock Docking score (kJ/mol)** | **Predicted Autodock inhibitory constant value (µM), ^*^Experimental IC_50_; µM** |
| --- | --- | --- | --- |
| Alectinib | -30.32 | -32.76 | 2.97 |
| Crizotinib | -28.53 | -30.54 | 45.69 |
| Erlotoinib | -25.22 | -29.70 | 120.43 |
| Geftinib | -25.67 | -28.45 | 122.33 |
| Lapatinib | -22.21 | -21.44 | 150 |
| Lorlatinib | -19.42 | -15.21 | 187.32 |

**Molecular Dynamic simulations**

**RMSD analysis of NEK7-Alectinib complex**

In order to validate the docking results, MD simulations were performed in triplicate. The main production run was performed for 200 ns, and the other two were performed for 50 ns. It was observed that the second and third production runs showed RMSD patterns ranging from 4 to 5 angstroms. The average RMSD value for the second run was 4 angstroms, and the trajectory was quite stable and equilibrated. Moreover, production run 3 demonstrated a similar RMSD pattern. It was observed that RMSD values fluctuate around 4 angstrom, and the average RMSD value was 4.6 angstrom. Hence, it is validated that in every simulation run, the RMSD pattern remained well below 5 angstroms. These patterns of RMSD validate the molecular docking results. Figure S1 and S2 show the evolution of the RMSD pattern for the Alectinib-NEK7 complex.


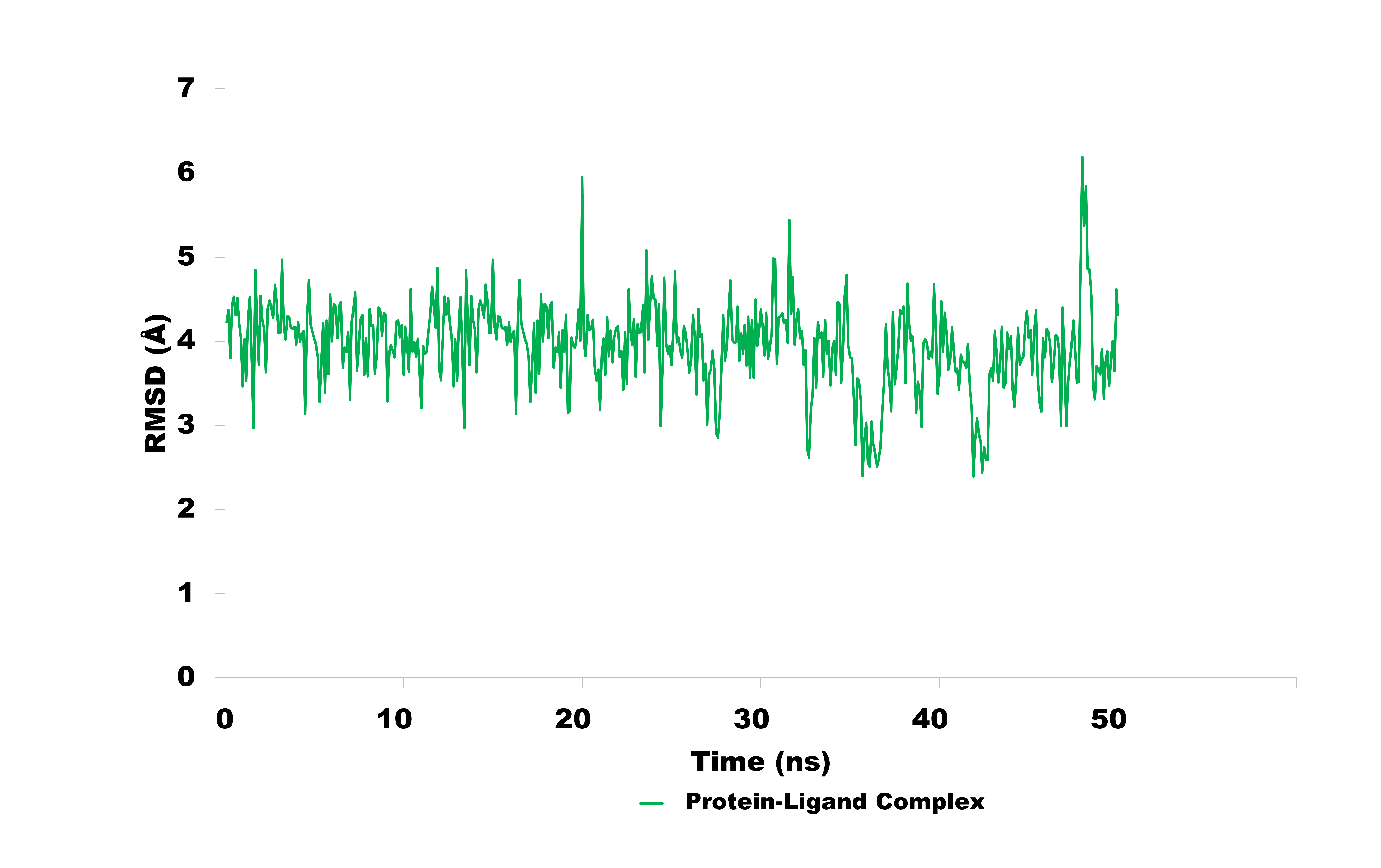


**Figure S1:** Production run 2 showing the evolution of RMSD pattern


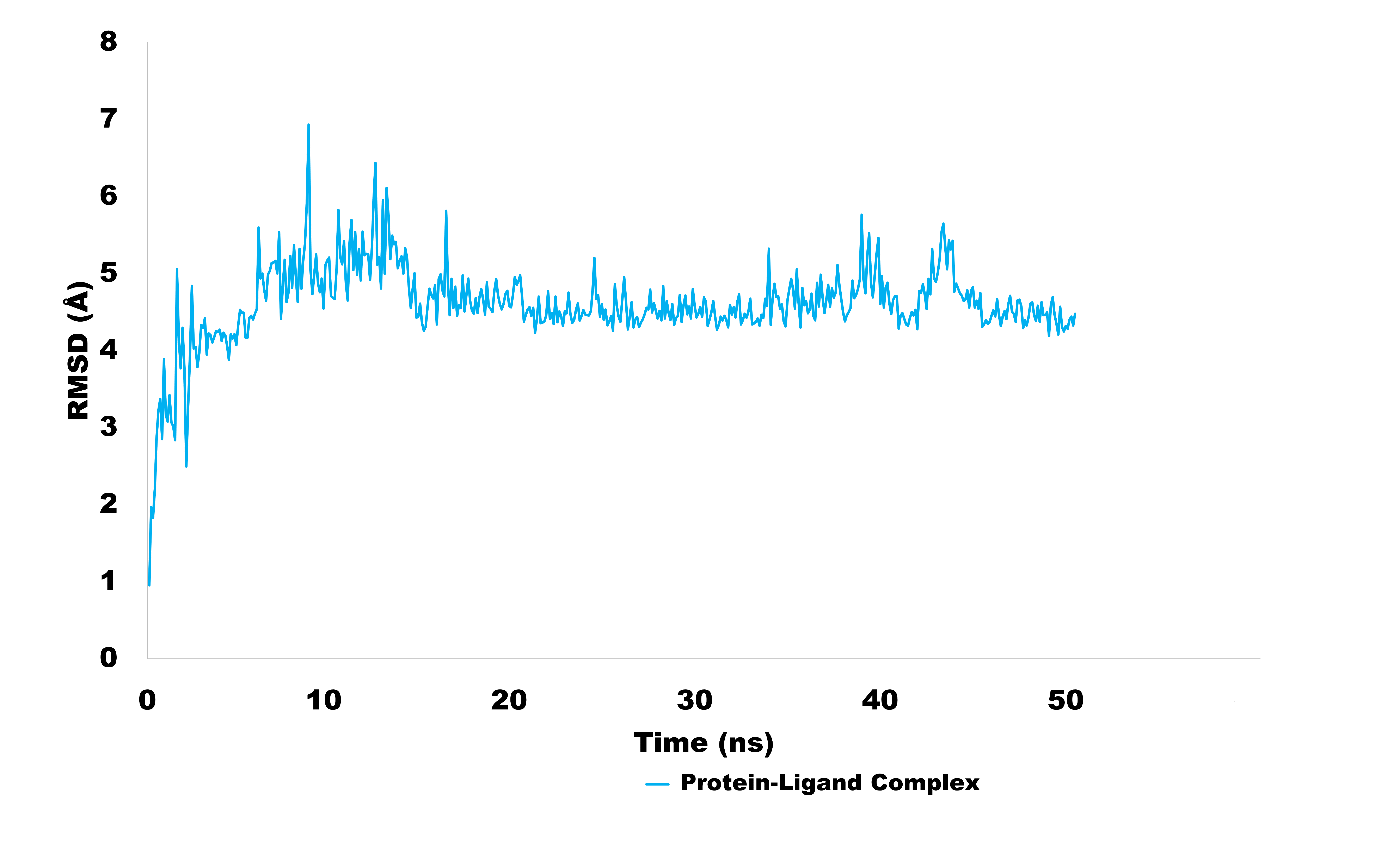


Figure S2: Production run 3 showing the evolution of RMSD pattern for NEK7-Alectinib complex

The secondary structure elements (SSE) like alpha-helices and beta strand are monitored during the simulation. The graph below depicts the distribution of SSE (figure S3) by residue index across the protein structure. Throughout the simulation, figure below showing the SSE elements with respect to individual frame and simulation time.

**
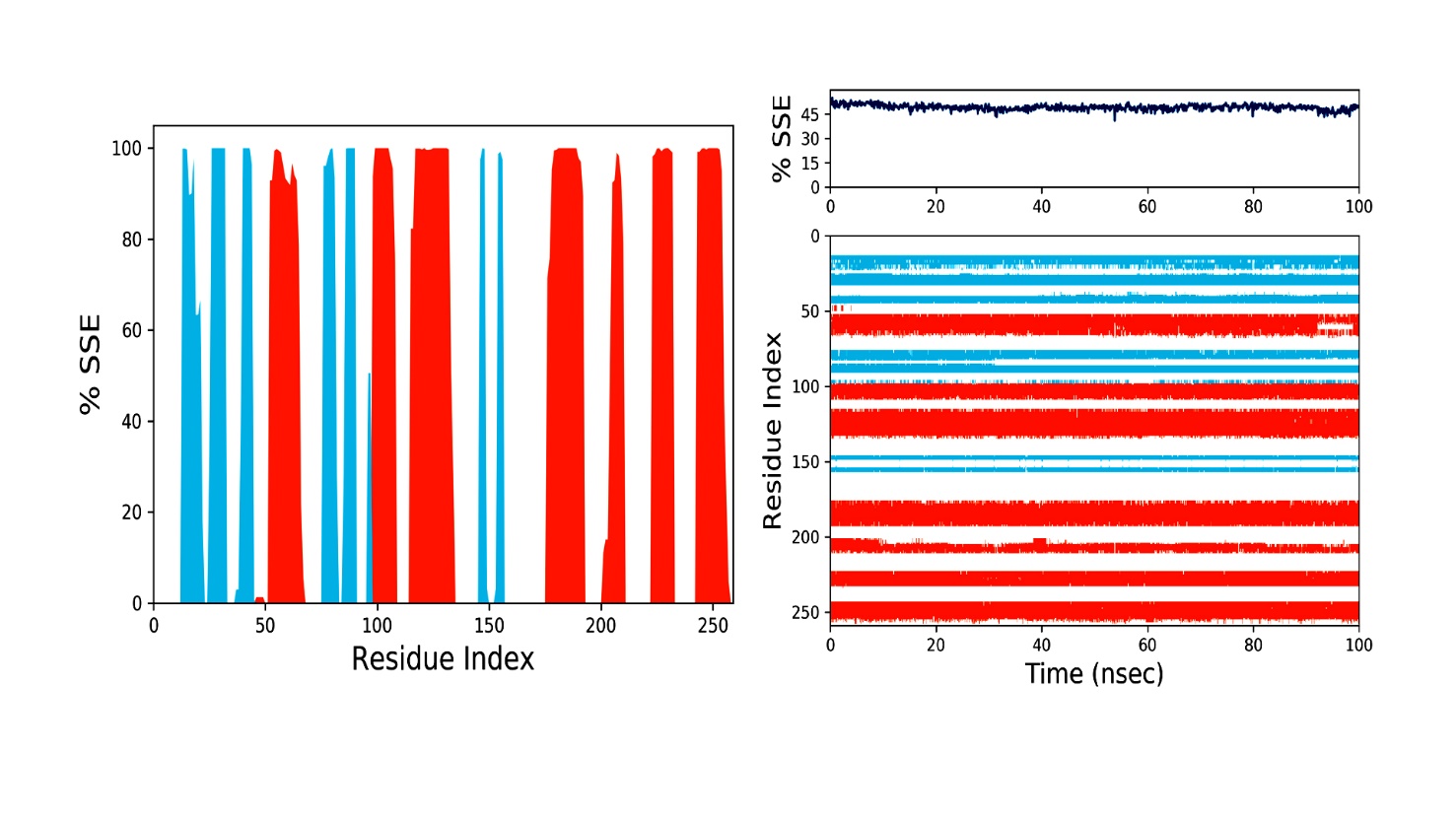
**

**Figure S3**. A) Secondary structural elements (SSE) of proteins, such as alpha-helices and beta-strands, were seen throughout the simulation.  The graph depicts the SSE distribution by residue index over the whole protein structure.  The backbone of the reference frame was used to align all of the protein frames. Energy efficiency was improved to reduce inappropriate geometry and avoid steric collisions. The protein's tails (both N and C-terminal) are known to fluctuate more than any other part of the protein. Secondary structural elements like alpha helices (Red) and beta strands (blue) are stiffer than the unstructured region of the protein and so change less than loop portions. B) The SSE composition for each trajectory frame throughout the run is shown in this graphic (simulation step).

The ligand torsions plot (Figure S4) depicts the structural evolution of each rotatable bond (RB) in the ligand during the simulation trajectory (0.00 through 100.00 ns). The top panel shows a two-dimensional schematic of a ligand with color-coded rotatable bonds. Each rotatable bond torsion is accompanied by a dial plot and bar plots of the same color.

**
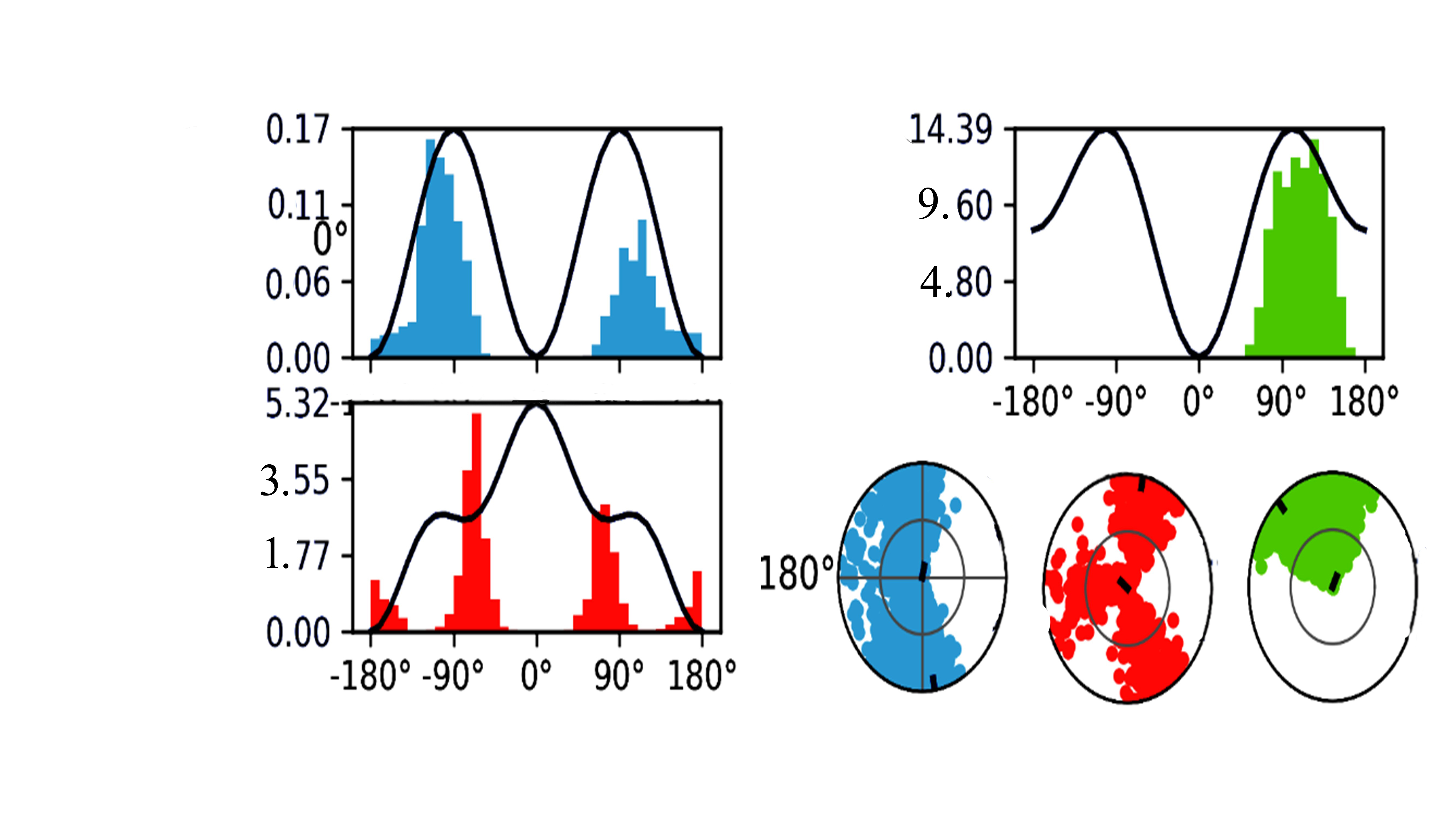
**

**Figure S4.** Within the NEK7-Alectinib torsion structure, simulation trajectory reveals rotatable links (0.00 through 100.00 nsec). The shape of the torsion during the simulation is represented by dial (or radial) graphs. Beginning in the center of the radial display, the simulation moves radially outward. In the bar charts that summaries the data from the dial plots, the probability density of torsion is represented. If torsional potential information is provided, the diagram also depicts the bond's potential (by summing the potentials of the related torsions). The potential values are shown on the left Y-axis of the graph and are given in kcal/mol. The connections between the histogram and torsion potential may reveal the conformational strain that the ligand must endure to maintain its protein-bound shape.

Ligand: Alectinib Profile

Individual interactions for Alectinib with NEK7 protein are shown in Figure 12. Drug interactions that last for more than 30.0 percent of the simulation time in the chosen trajectory (0.00 through 100.0 ns) are depicted.


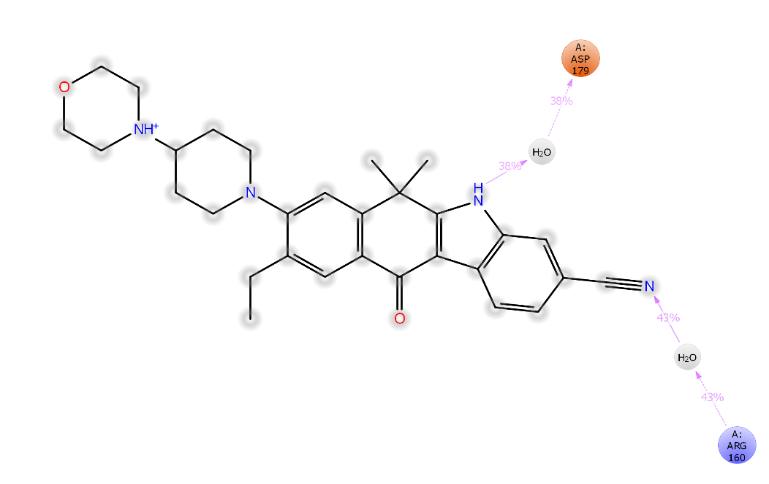


**Figure S5**. Depiction of ligand atom interactions with the NEK7-Alectinib. Detailed analysis showed those interactions that last more than 40% of the simulation time i.e. molecular interactions with residuesASP169 (hydrogen bonding) and ARG160 (water bridge).

The gyration radius (Rg) of a ligand and a protein is illustrated in Figure 13. (A, B). Rg quantifies the 'extensibility' of a ligand, which is proportional to its primary moment of inertia. Rg for ligand structure remained compact throughout the simulated trajectory, while Rg for protein initially demonstrated compactness, but after 50 ns protein exhibited structural variation in polar solvent. In addition, intramolecular hydrogen bonds refer to the number of internal hydrogen bonds (HB) inside a ligand molecule (intraHB). The chosen drugs were found to be devoid of intraHB. Additionally, Solvent Accessible Surface Area (SASA) refers to the surface area of a molecule that is accessible to a nearby solvent. SASA values remained between 448 and 460 Å2, indicating that Alectinib was optimally exposed to the surrounding solvent. The polar surface area (PSA) of a molecule is the region where oxygen and nitrogen atoms may be accessed by a solvent on their own. Figure 13 (A) illustrates the detailed ligand characteristics, whereas Figure 13 (B) depicts the radius of gyration of the NEK7 protein.


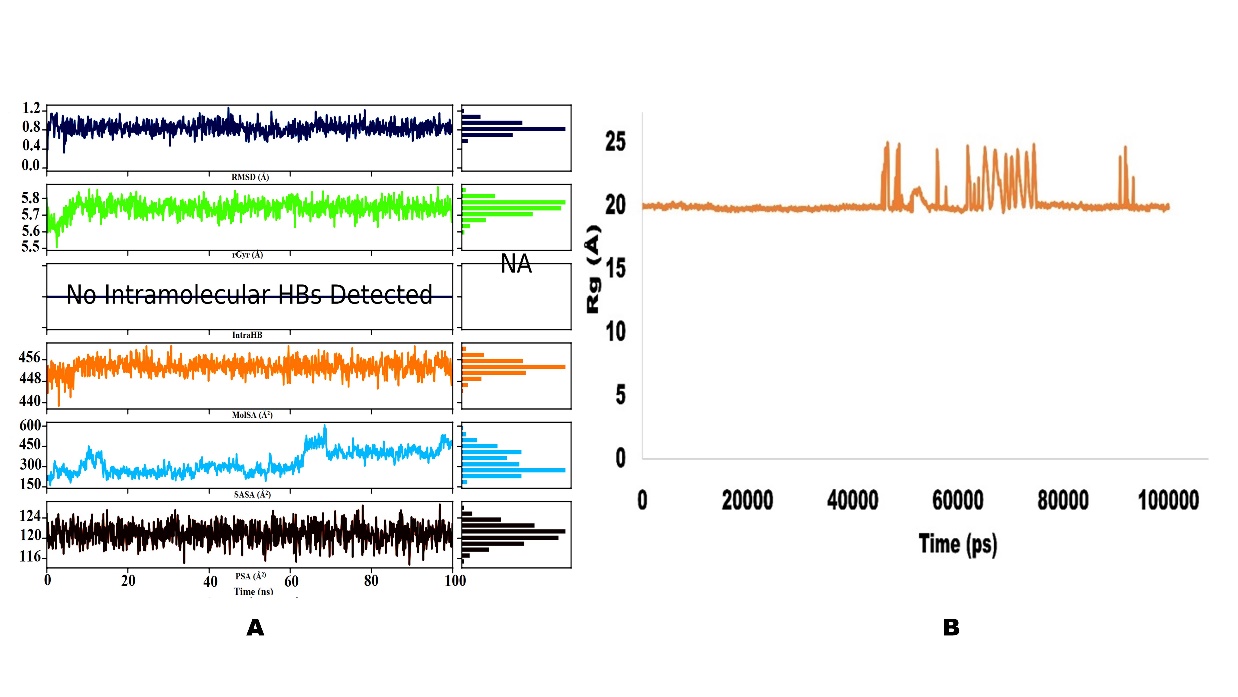


**Figure S6.** A) Ligand properties including RMSD (Å), MolSA (Å2), Rg (Å), SASA (Å2) and PSA (Å2) B). Radius of Gyration of NEK7 protein.

**MD simulation of NEK7-Crizotinib complex**

**RMSD and RMSF analysis of NEK7-Crizotinib complex**

The 2WQN-Crizotinib complex's RMSD plot (Figure S5) shows that the complex showed stability initially but start fluctuating after 15 ns. After 30 ns, RMSD of complex was dropped below 2 angstrom and remained stable for 60ns of simulation. After that complex was fluctuated to 2.8 angstrom which was again got stable around 2.1 angstrom after 80 ns which is perfectly acceptable. After being equilibrated, protein RMSD values remained stable throughout the trajectory. Between 60 to 80 Angstrom, its RMSD was slightly higher. Then for the period of 90 ns its RMSD decreased and remained stable for remaining period of simulations.


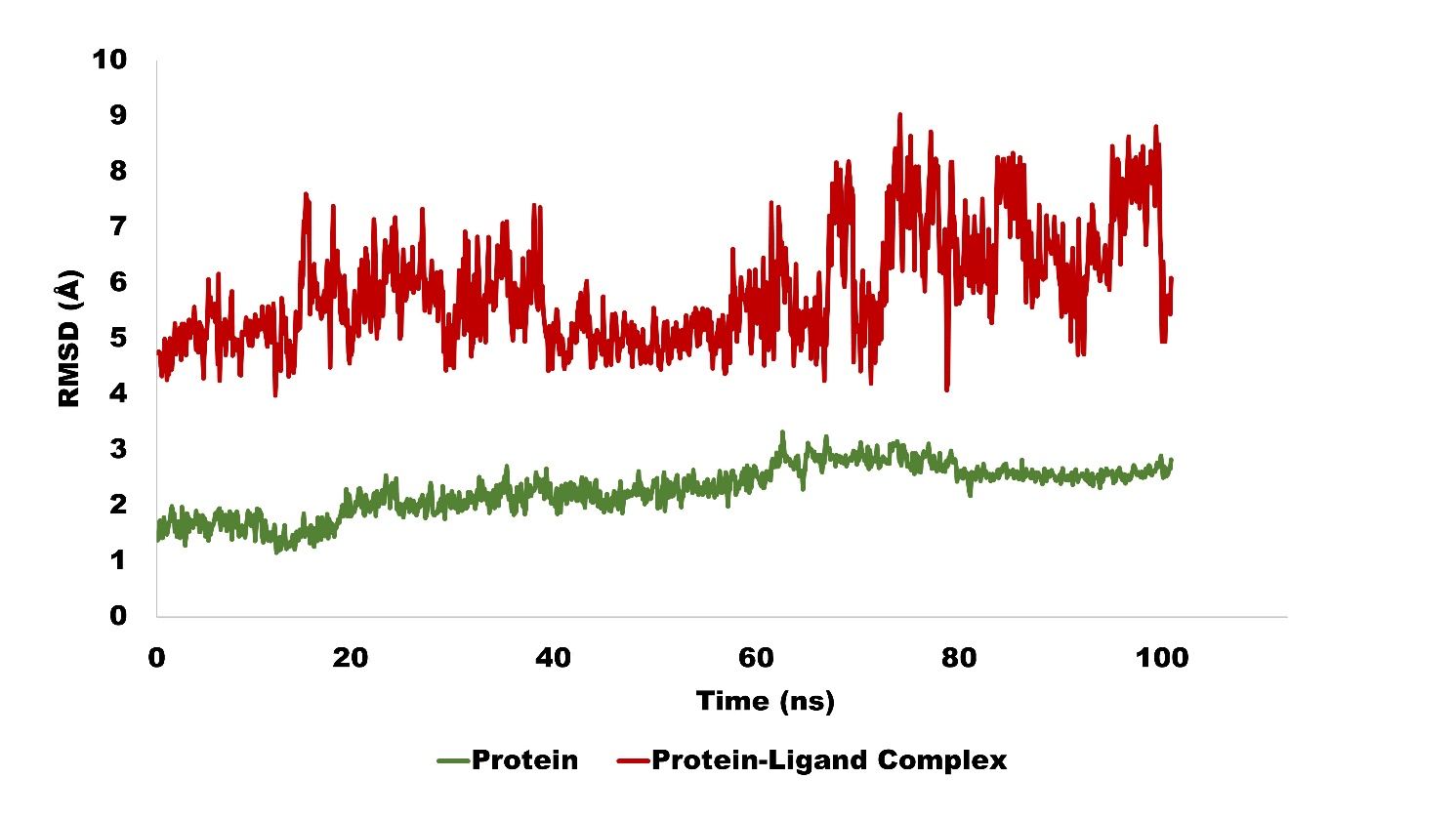


**FigureS7**. Residue wise Root mean square deviation (RMSD) of the C-alpha atoms of NEK7 (2WQN) and Crizotinib Complex with time. The left Y-axis depicts the change in protein RMSD over time. The right Y-axis depicts the change in ligand RMSD over time.

Protein-ligand showed potent interactions during course of simulation. This interaction included hydrogen bonding, hydrophobic, ionic bonds and water bridges. Figure S6 showing the different types of interactions. In case of NEK7-Crizotinib complex, ASP179 and ALA114 were important residues in terms of hydrogen bonding. Moreover, stacked bar chart demonstrating the time of contact maintained over the course of simulation time. A value of 1 showed that contact is maintained for 100% simulation time. Values above 1.0 are feasible.


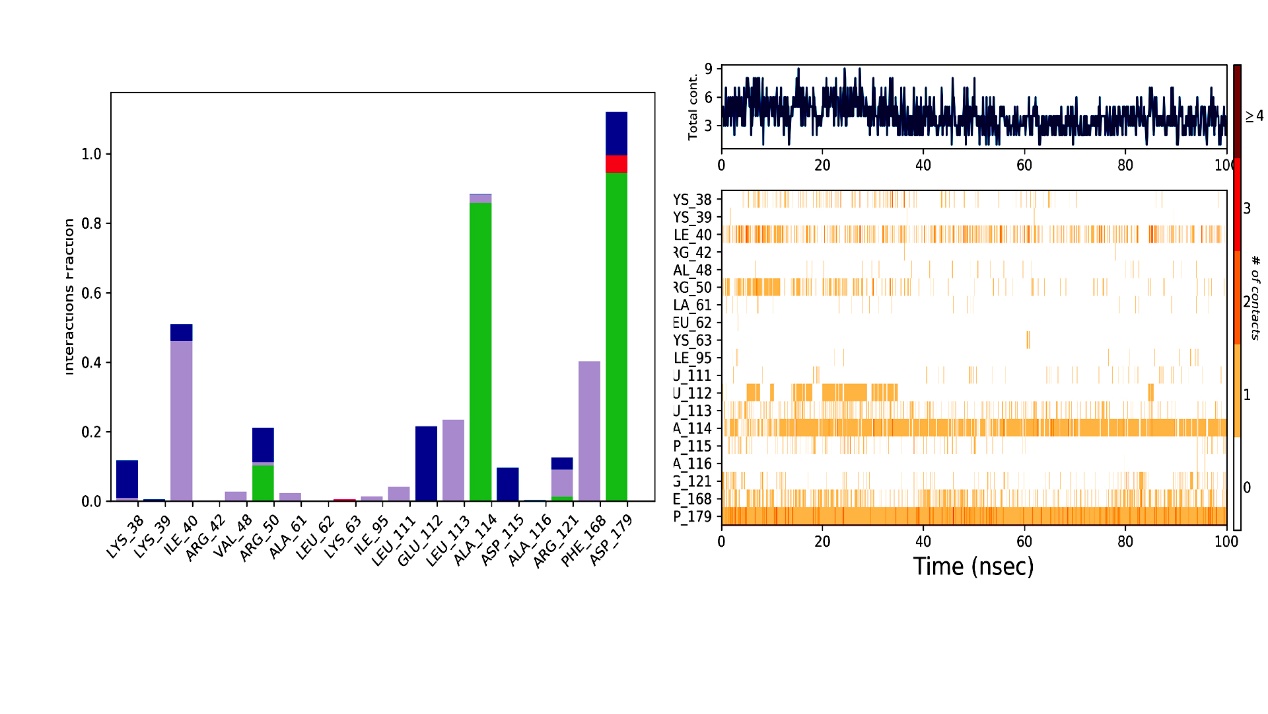


**FigureS8.** Protein-ligand (NEK7-Crizotinib) contact histogram (H-bonds, Hydrophobic, Ionic, Water bridges).

**Ligand: Crizotinib Profile**

Individual interactions for Crizotinib with NEK7 protein are shown in Figure S7 Drug interactions that last for more than 30.0 percent of the simulation time in the chosen trajectory (0.00 through 100.0 ns) are depicted.


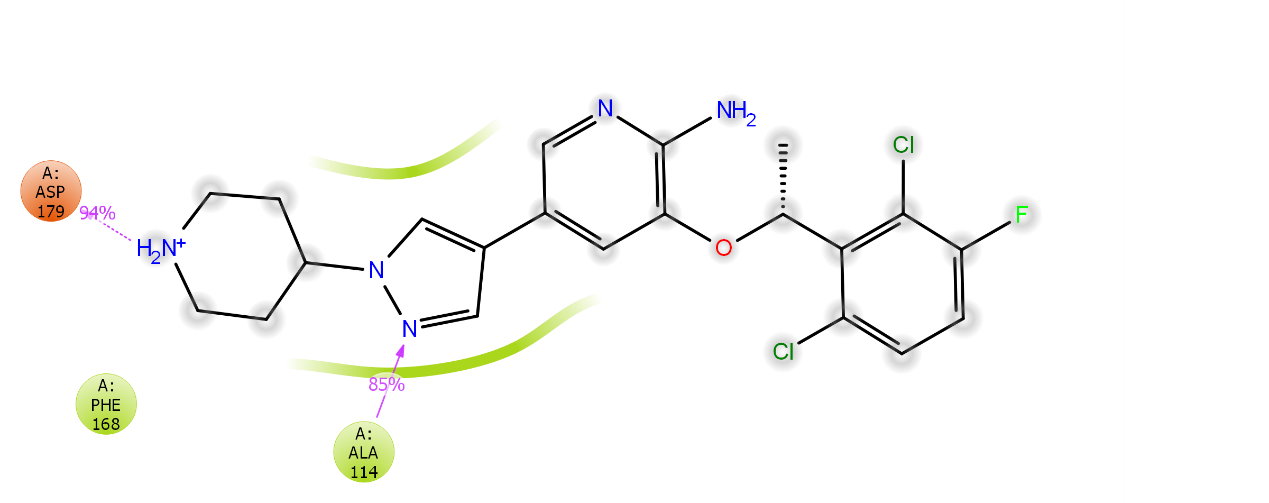


**FigureS9**. Ligand atom interactions with the protein residues. (NEK7-Crizotinib)

Ligand RMSD with respect to the reference conformation (usually the first frame, which is regarded as time t=0) rGyr (radius of gyration, Figure S8). Measures a ligand's 'extendedness,' which is the same as its primary moment of inertia.


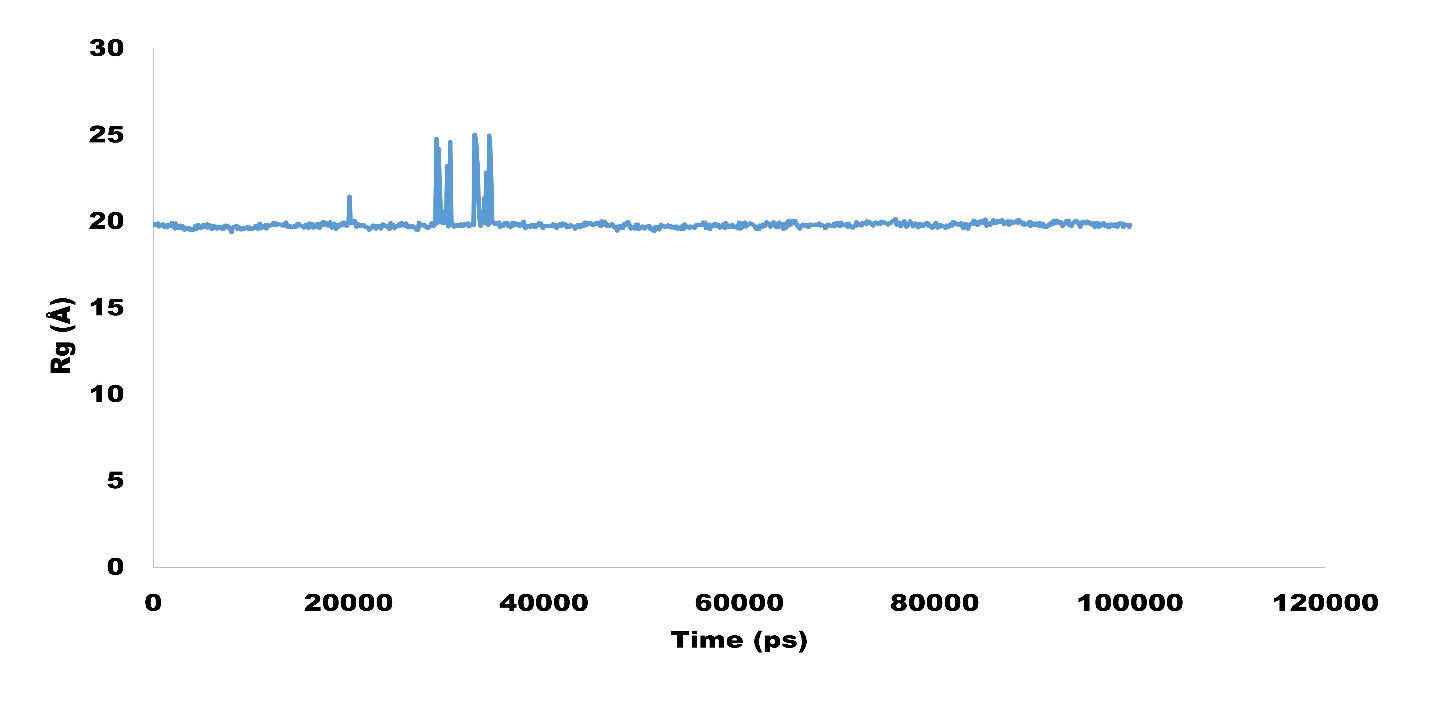


**Figure S10.** Radius of Gyration of protein bound to ligand

Throughout the simulation trajectory, the ligand torsions plot describes the conformational evolution of each rotatable bond (RB) in the ligand (0.00 through 100.00 ns). The top panel depicts a two-dimensional schematic of a ligand with rotatable bonds that are color-coded. A dial plot and bar plots of the same color accompany each rotatable bond torsion (Figure S9).


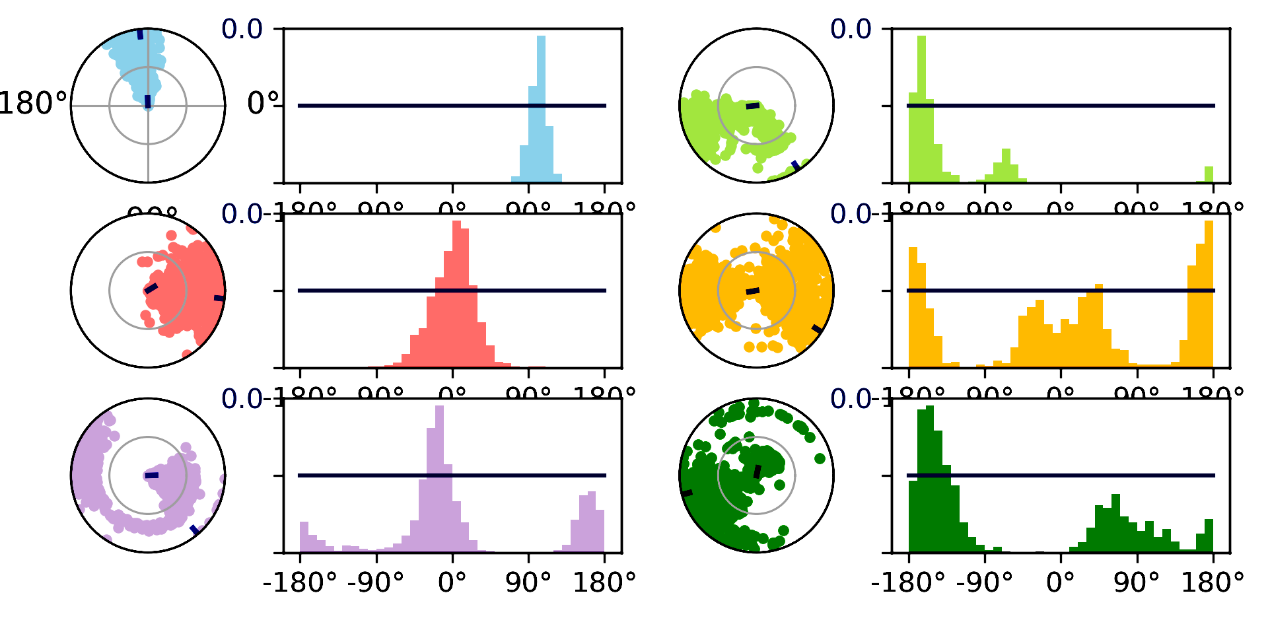


**Figure S11**. Ligand Torsion Profile

Dial (or radial) plots depict the torsion's conformation during the course of the simulation (Figure S10). The simulation starts at the center of the radial display, and the time evolution is plotted outwards radially. The probability density of the torsion is shown in the bar charts, which summaries the data from the dial plots. If torsional potential data is provided, the graphic additionally shows the rotatable bond's potential (by summing the potential of the related torsions). The potential values are displayed on the chart's left Y-axis and are given in kcal/mol. The histogram and torsion potential correlations can reveal the conformational strain that the ligand goes through to maintain a protein-bound shape.


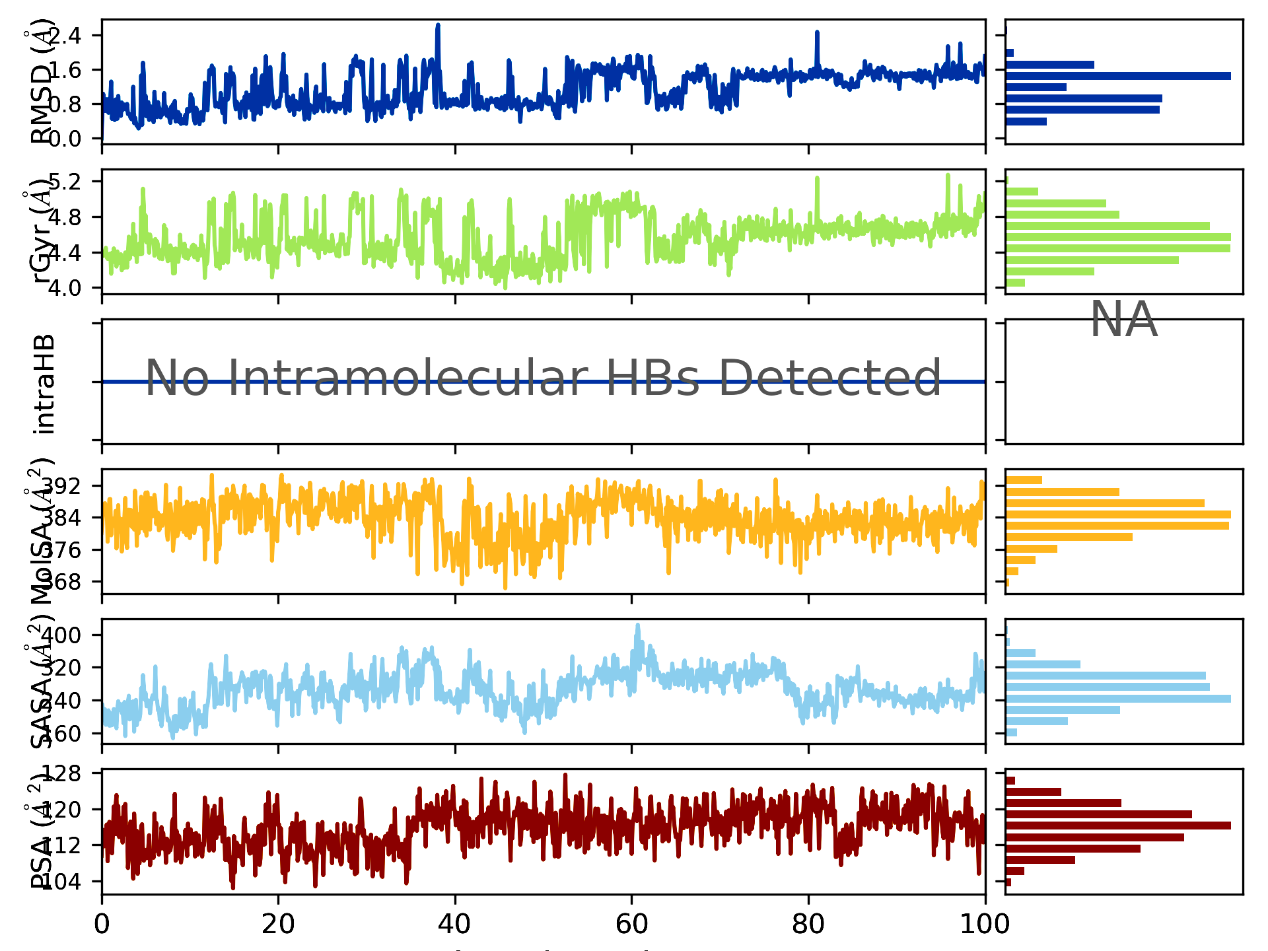


**Figure S12**. Ligand properties

**Normal mode analysis (NMA) of Apo protein and Liganded protein**

In addition to the deformability and covariance analysis, additional structural analysis methods such as B-factor, elastic network, variance, and eigenvalue analysis were performed for the apo NEK7 protein, the NEK7-alectinib complex, and the NEK7-compound 146476703 complex.

The B-factor analysis provides information about the flexibility of individual residues in a protein. The results showed that the apo NEK7 protein had high flexibility in certain regions, as indicated by high B-factors. However, the formation of complexes with alectinib and compound 146476703 led to a decrease in the overall flexibility of the protein, as indicated by lower B-factors. This suggests that the presence of these molecules may lead to the restriction of movements in certain regions of the protein.


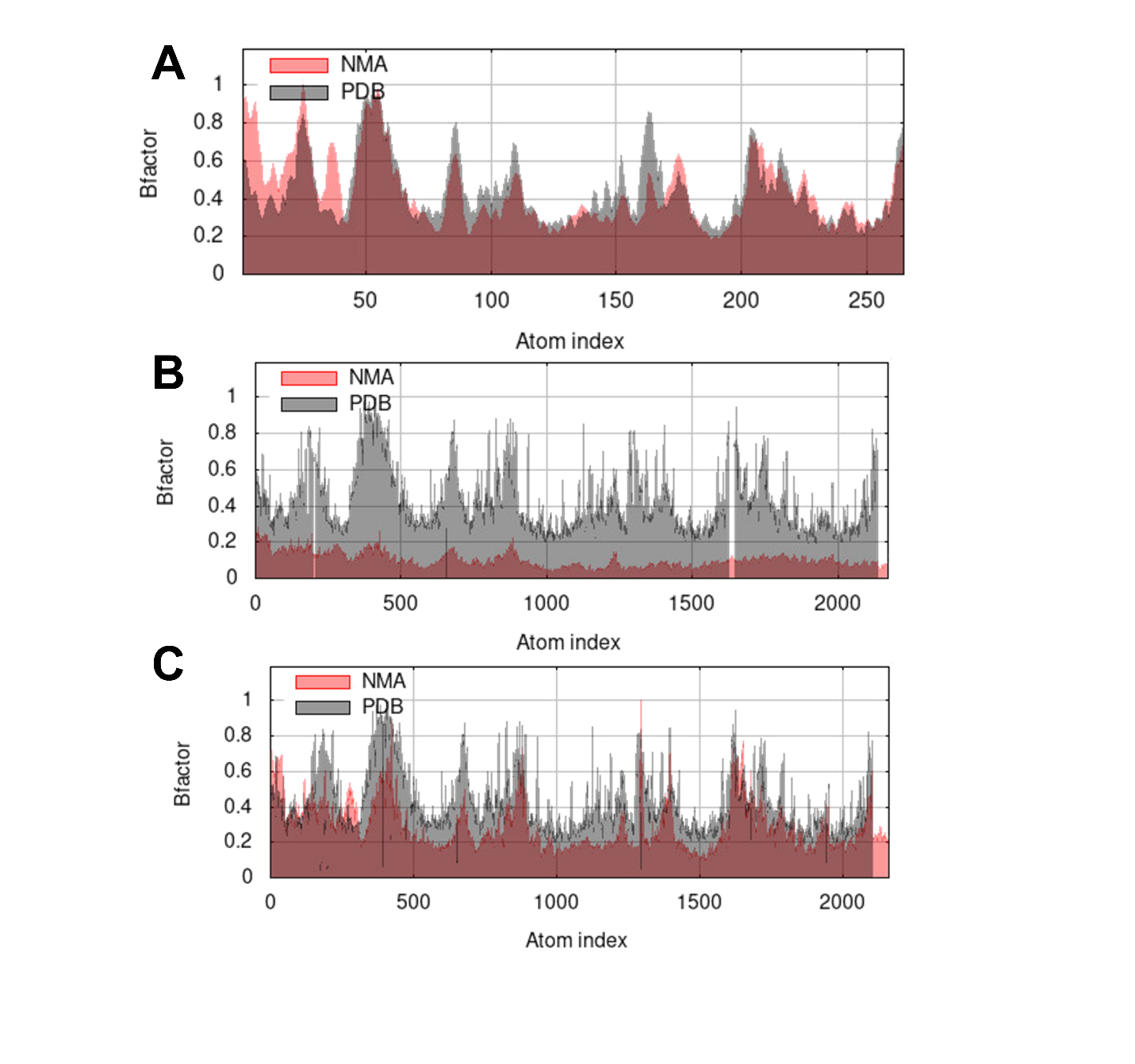


**Figure S13.** B factor analysis of Apo protein (A), NEK7-alectinib complex (B) and NEK7 and compound 146476703 complex (C)

The elastic network analysis provides information about the collective motions of residues in a protein. The results showed that the apo NEK7 protein had a relatively loose elastic network, with many residues exhibiting a high degree of freedom in their movements. However, the formation of complexes with alectinib and compound 146476703 led to a tightening of the elastic network, as indicated by a decrease in the overall motion of residues. This suggests that the presence of these molecules may lead to the restriction of movements in the protein as a whole.


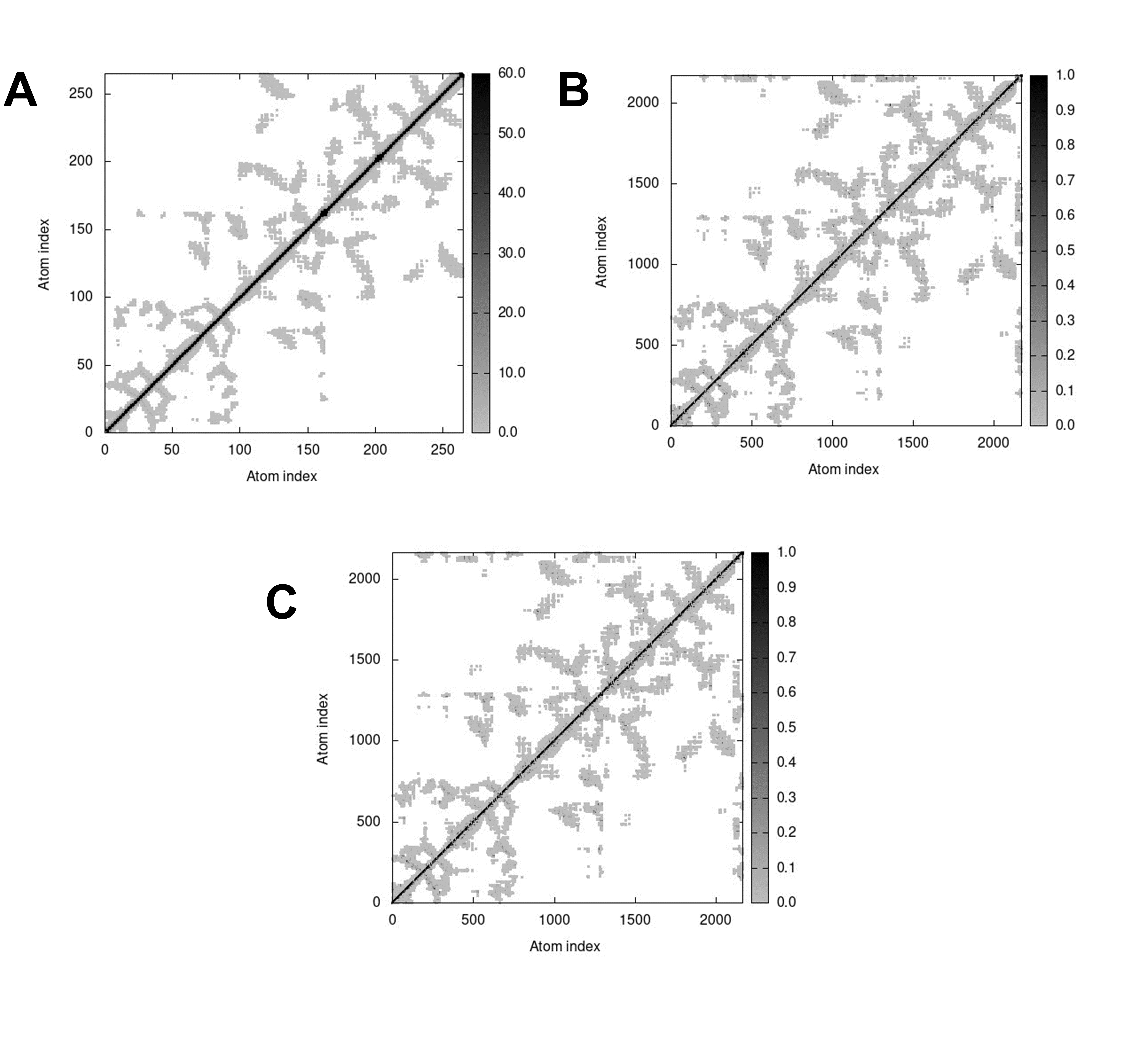


**Figure S14.** Elastic map network of Apo protein (A), NEK7-alectinib complex (B) and NEK7 and compound 146476703 complex (C)

The variance and eigenvalue analysis provide information about the distribution of motions in a protein. The results showed that the apo NEK7 protein had a relatively high degree of variation in the motions of its residues, as indicated by high variances and eigenvalues. However, the formation of complexes with alectinib and compound 146476703 led to a decrease in the overall variation of motions, as indicated by lower variances and eigenvalues. This suggests that the presence of these molecules may lead to the restriction of movements in the protein, leading to a more structured and rigid state.


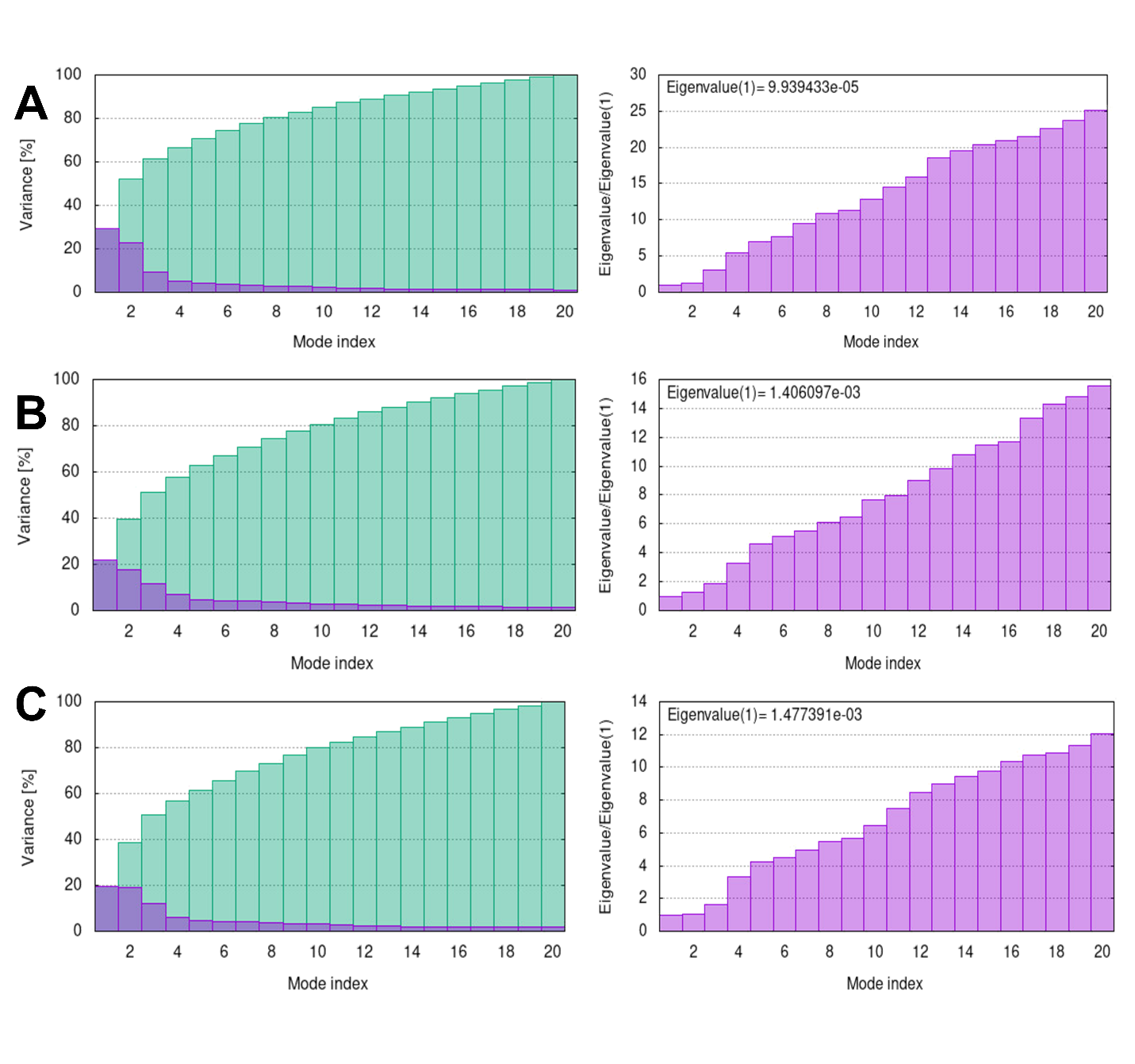


**Figure S15.** Eigenvalue and variance of Apo protein (A), NEK7-alectinib complex (B) and NEK7 and compound 146476703 complex (C)

**Molecular docking Studies against NEK2**

Yes reviewer is right about the structural similarities among the NEK family members. Here is the justification of the selectivity of the inhibitors more towards NEK7. The compounds were docked within the active pocket of NEK2 (PDB ID: 5M57; resolution 2.30Å) and selectivity was found comparatively low and it can be suggested that in comparison to NEK7 protein, docked conformations of compound Alectinib, Crizotinib, Erlotinib and Geftinib within the active pocket of NEK2 protein exhibited less stable interactions; notably, notably, these drugs were slightly protruded out from the NEK2 protein's activation loop. Alectinib, Crizotinib, Erlotinib and Geftinib docking scores were -18.3, -13.4, 13.6 and -11.6 kJ/mol, respectively. Unfavorable / bumping interactions were also observed in case of NEK2 and all these ligands also showed interactions with the amino acid residues other than active pocket. (Figure given below).


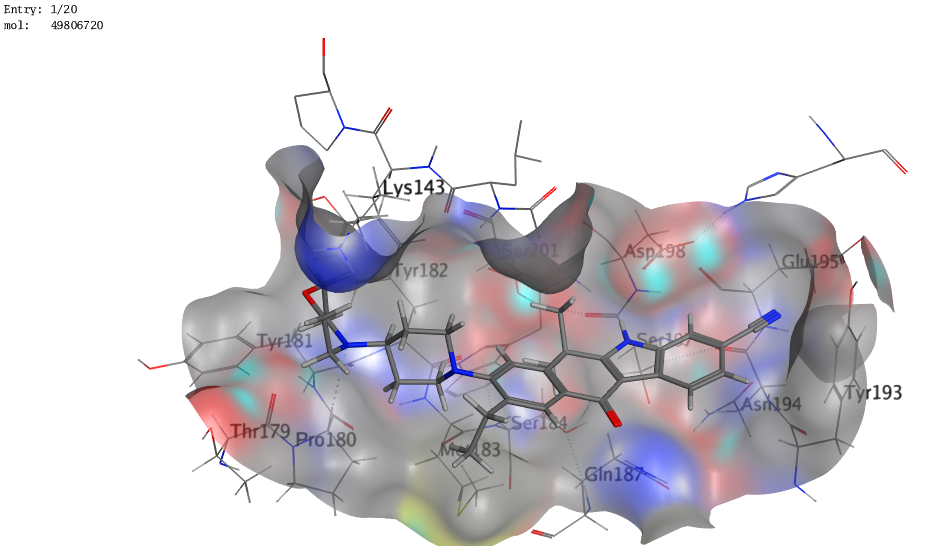

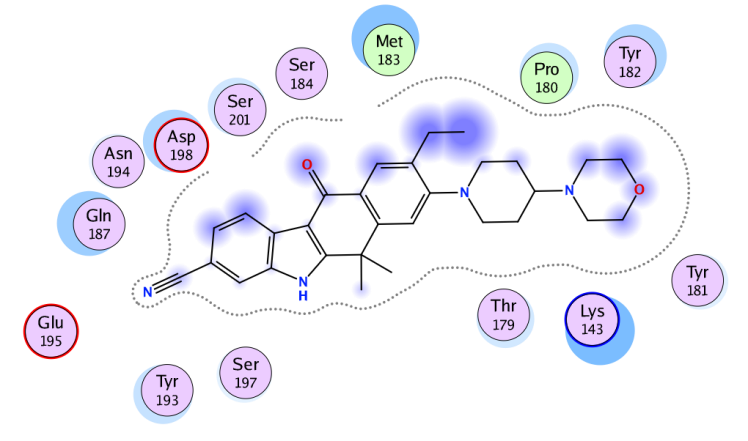


**Figure S16**. 2D and 3D interactions of NEK2-Alectinib complex (Molecular Operating Environment (MOE) 2015.10 [32])


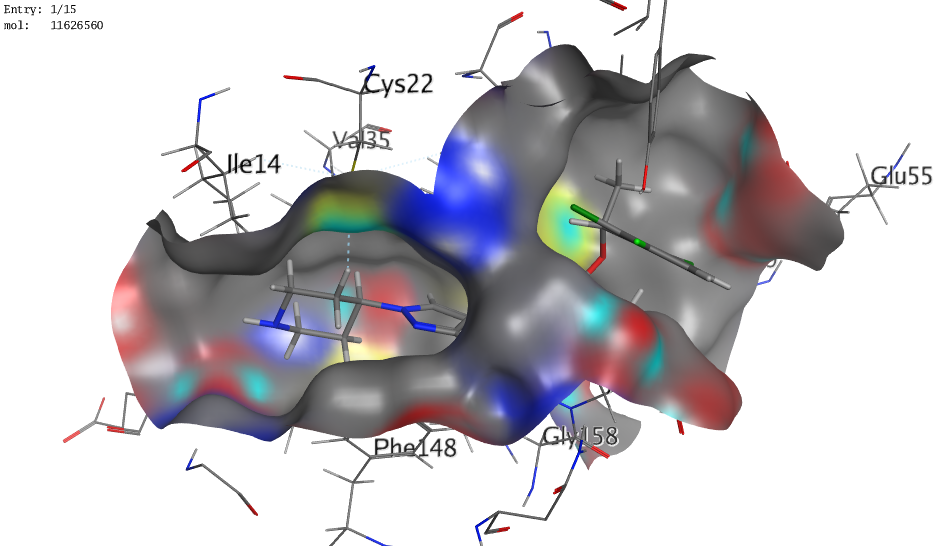

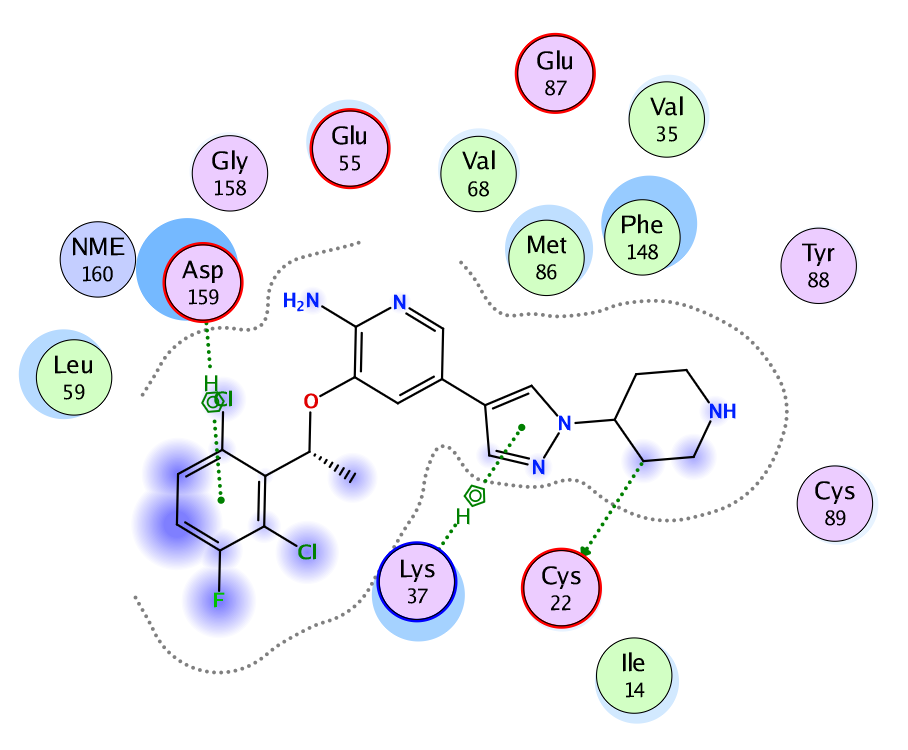


**Figure S17.** 2D and 3D interactions of NEK2-Crizotinib complex (Molecular Operating Environment (MOE) 2015.10 [32])


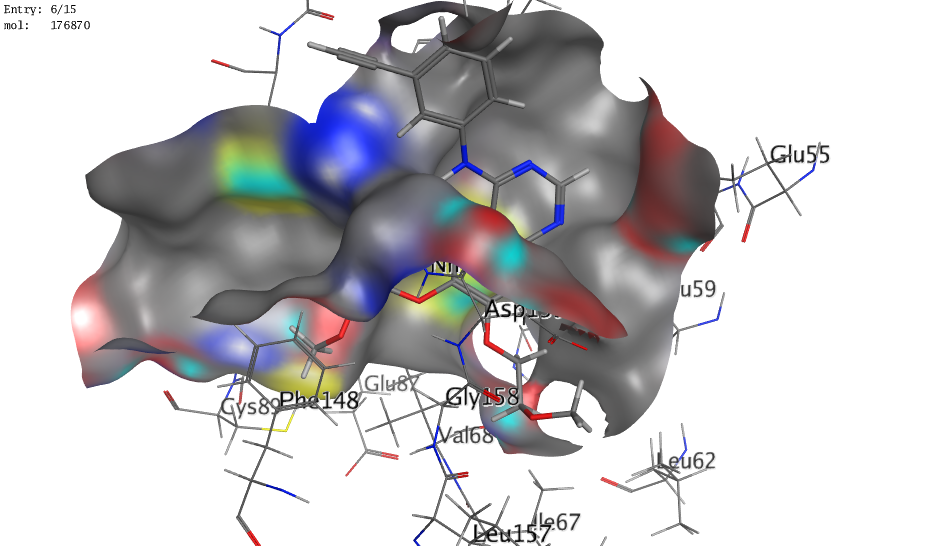

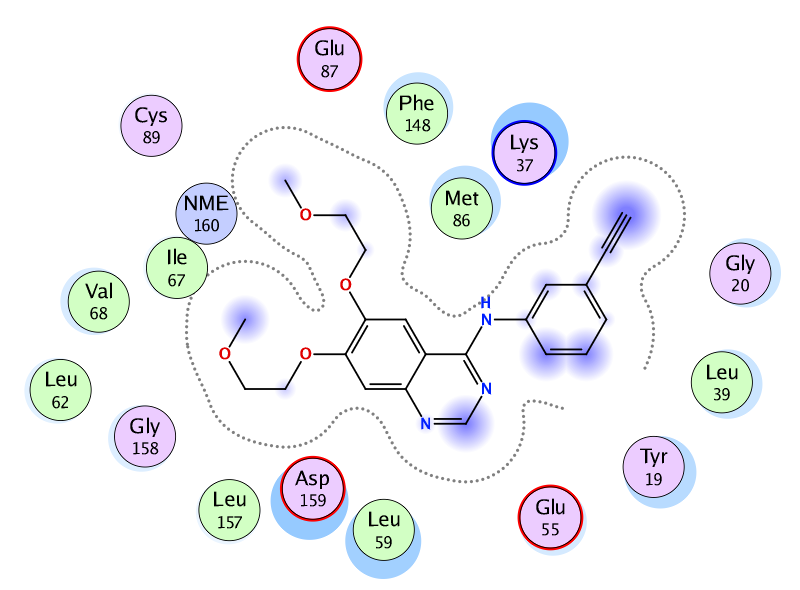


**Figure S18**. 2D and 3D interactions of NEK2-Erlotinib complex (Molecular Operating Environment (MOE) 2015.10 [32])


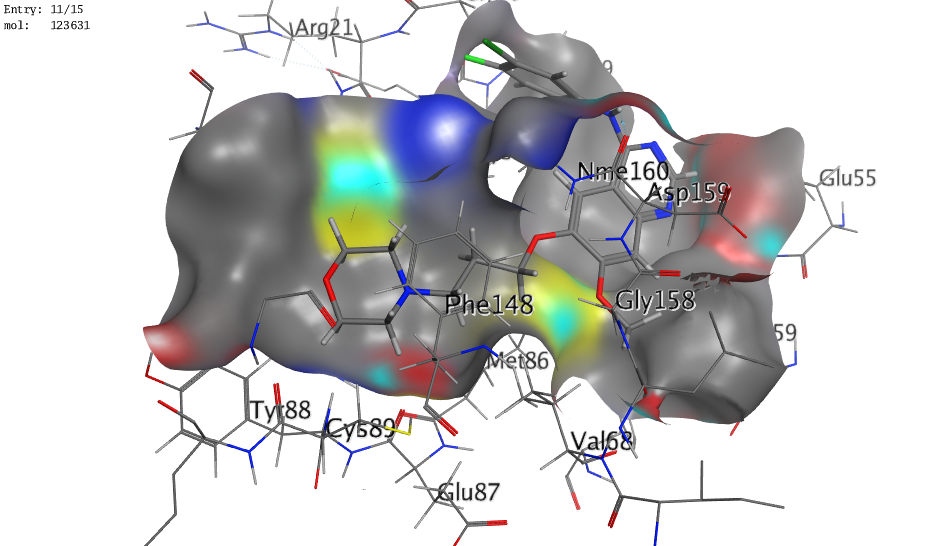

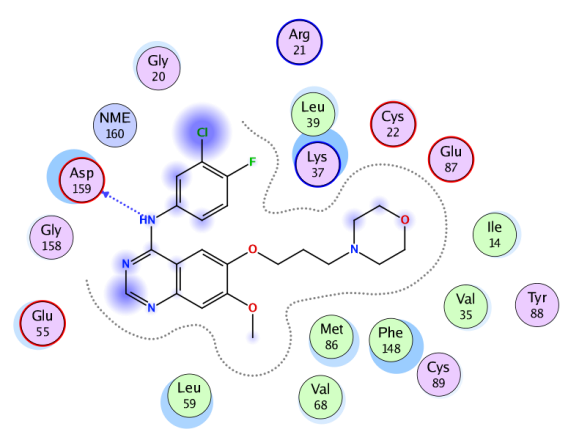


**Figure S19**. 2D and 3D interactions of NEK2-geftinib complex

Table S2. Hydrogen bonding observed during Molecular docking studies with NEK2

| **Compound** | **Residue** | **Distance**  **H-A** | **Distance**  **D-A** | **Angle** | **Side chain** | **Donor atom** | **Acceptor atom** |
| --- | --- | --- | --- | --- | --- | --- | --- |
| **Alectinib** | LYS143 | 2.18 | 3.79 | 132.50 |  | -- | -- |
| **Crizotinib** | CYS89 | 3.09 | 3.69 | 122 | -- | -- | 1443 [O2] |
| **Erlotinib** | ASP159 | 2.83 | 3.57 | 131.04 | -- | -- | 4095 [O3] |
| **Gefitinib** | GLU55, ASP159 | 3.12, 3.39 | 3.36, 4.09 | 149.31, 161.31 | - ,-- | -- | 844 [O.co2], 4098 [O3] |

Table S3. Hydrophobic interactions observed with NEK2

| **Compound** | **Interaction** | **Residue** | **Distance** | **Angle** | **Ligand atom** |
| --- | --- | --- | --- | --- | --- |
| **Alectinib** | Hydrophobic | ILE40, VAL48, PHE168, ASP115 | 3.71 | -- | --- |
| **Crizotinib** | Hydrophobic, Halogen Bond, Salt Bridges | PHE148, ASP159, GLU55, ASP159 | 3.12, 3.78, 4.85 | -- | --- |
| **Erlotinib** | Hydrophobic, Salt Bridges | LYS37, VAL68, ASP159, GLU55 | 3.73, 5.27 | -- | --- |
| **Gefitinib** | Hydrophobic, Salt Bridges | LYS37, LEU59, VAL68, ASP159, GLU55 | 3.77, 3.66, 3.96, 3.86, 4.55 | -- | --- |

**Density Functional theory studies (DFTs)**

**Bond length and Bond angle**

Structural data of optimized drugs showed that longest bond length was 1.755 angstrom observed between C27-C129 present in Crizotinib. Whereas shortest bond length was H37-N1 with bond length of 1.011 angstrom present in Alectinib. Similarly, bond angle of benzene ring was remained around 120 degrees which is perfect hexagonal geometry. However, substitution of nitrogen and oxygen atom cause reduction in bond length as both atoms are more electronegative than carbon atom. Structural data of optimized drugs i.e., alectinib, crizotinib, erlotinib and gefitinib is shown in table S4, S5, S6 and S7.

There are different types of bonds including C-N, C-O, N-N, C-C and C-H. The bond length and bond angles of all drugs are tabulated below.

**Table S4**: Optimized structural parameters of Alectinib at B3LYP/SVP.

| **Bond** | **Bond length (Å)** | **Bond** | **Bond angle (◦)** |
| --- | --- | --- | --- |
| N1-C2 | 1.373 | N1-C2-C7 | 108.984 |
| C2-C3 | 1.504 | C3-C4-C5 | 123.283 |
| C3-C4 | 1.539 | C6-C7-C2 | 122.559 |
| C4-C5 | 1.411 | C8-C9-C10 | 118.648 |
| C5-C6 | 1.494 | C9-C10-C11 | 121.008 |
| C6-C7 | 1.457 | C10-C11-C12 | 121.160 |
| C7-C8 | 1.439 | C11-C12-C13 | 117.170 |
| C8-C9 | 1.409 | C14-C15-C16 | 117.883 |
| C9-C10 | 1.390 | C15-C16-C17 | 118.229 |
| C10-C11 | 1.421 | C16-C17-C4 | 123.358 |
| C11-C12 | 1.406 | C17-C4-C5 | 117.943 |
| C12-C13 | 1.393 | C4-C5-C14 | 118.944 |
| C14-C15 | 1.392 | C5-C14-C15 | 123.492 |
| C15-C16 | 1.425 | N21-C22-C23 | 114.156 |
| C16-C17 | 1.410 | C22-C23-C24 | 111.465 |
| C18-N19 | 1.162 | C23-C24-C25 | 109.245 |
| C6-O20 | 1.226 | C24-C25-C26 | 111.499 |
| N21-C22 | 1.459 | C25-C26-N21 | 112.049 |
| C22-C23 | 1.538 | C26-N21-C22 | 111.811 |
| C23-C24 | 1.539 | N27-C28-C29 | 112.988 |
| C24-C25 | 1.544 | C28-C29-O30 | 110.548 |
| C25-C26 | 1.538 | C29-O30-C31 | 112.050 |
| N27-C28 | 1.455 | O30-C31-C32 | 111.001 |
| C28-C29 | 1.533 | C31-C32-N27 | 112.800 |
| C29-O30 | 1.416 | C32-N27-C28 | 111.687 |
| O30-C31 | 1.416 |  |  |
| C31-C32 | 1.534 |  |  |
| C3-C33 | 1.551 |  |  |
| C3-C34 | 1.551 |  |  |
| C15-C35 | 1.517 |  |  |
| C35-C36 | 1.536 |  |  |
| H37-N1 | 1.011 |  |  |
| H38-C9 | 1.090 |  |  |
| H39-C10 | 1.091 |  |  |
| H40-C12 | 1.091 |  |  |
| H41-C14 | 1.093 |  |  |
| H42-C17 | 1.088 |  |  |

**Table S5**: Optimized structural parameters of Crizotinib at B3LYP/SVP

| **Bond** | **Bond length (Å)** | **Bond** | **Bond angle (◦)** |
| --- | --- | --- | --- |
| C1-C2 | 1.419 | C1-C2-C3 | 118.938 |
| C2-C3 | 1.384 | C2-C3-C4 | 119.985 |
| C3-C4 | 1.404 | C3-C4-C5 | 116.118 |
| C4-C5 | 1.404 | C4-C5-N6 | 124.478 |
| C5-N6 | 1.335 | C5-N6-C1 | 119.004 |
| N6-C1 | 1.306 | N6-C1-C2 | 121.424 |
| C7-C8 | 1.439 | N13-C14-C15 | 109.790 |
| C1-N7 | 1.381 | C14-C15-C16 | 110.180 |
| N8-N9 | 1.343 | C15-C16-C17 | 110.580 |
| N9-C10 | 1.329 | C16-C17-C18 | 110.109 |
| C10-C11 | 1.424 | C17-C18-N13 | 109.730 |
| C11-C12 | 1.393 | C18-N13-C14 | 112.641 |
| N13-C14 | 1.457 | N8-N9-C10 | 105.329 |
| C14-C15 | 1.531 | N9-C10-C11 | 111.961 |
| C15-C16 | 1.536 | C10-C11-C12 | 103.321 |
| C16-C17 | 1.535 | C11-C12-N8 | 107.147 |
| C17-C18 | 1.531 | C12-N8-N9 | 112.236 |
| C18-N13 | 1.457 | C2-O19-C20 | 114.543 |
| O19-C20 | 1.438 | O19-C20-C21 | 107.966 |
| C20-C21 | 1.523 | C22-C23-C24 | 121.240 |
| C20-C22 | 1.528 | C23-C24-C25 | 120.883 |
| C22-C23 | 1.411 | C24-C25-C26 | 119.114 |
| C23-C24 | 1.402 | C25-C26-C27 | 120.067 |
| C24-C25 | 1.388 | C26-C27-C22 | 122.164 |
| C25-C26 | 1.393 | C27-C22-C23 | 116.523 |
| C26-C27 | 1.398 |  |  |
| C27-C22 | 1.410 |  |  |
| C23-C130 | 1.747 |  |  |
| C27-C129 | 1.755 |  |  |

**Table S6**: Optimized structural parameters of Erlotinib at B3LYP/SVP

| **Bond** | **Bond length (Å)** | **Bond** | **Bond angle (◦)** |
| --- | --- | --- | --- |
| N1-C2 | 1.314 | N1-C2-N3 | 127.350 |
| C2-N3 | 1.349 | C2-N3-C4 | 117.858 |
| N3-C4 | 1.326 | N3-C4-C5 | 121.003 |
| C4-C5 | 1.437 | C4-C5-C10 | 115.128 |
| C5-C10 | 1.429 | C5-C10-N1 | 122.081 |
| C10-N1 | 1.362 | C10-N1-C2 | 116.112 |
| C7-C8 | 1.439 | C5-C6-C7 | 120.861 |
| C5-C6 | 1.420 | C6-C7-C8 | 119.822 |
| C6-C7 | 1.382 | C7-C8-C9 | 119.511 |
| C7-C8 | 1.448 | C8-C9-C10 | 121.527 |
| C8-C9 | 1.379 | C9-C10-C5 | 118.589 |
| C9-C10 | 1.420 | C10-C5-C6 | 119.433 |
| C10-C5 | 1.429 | C4-C11-N22 | 128.877 |
| C4-N11 | 1.384 | C22-C23-C24 | 120.889 |
| N11-C22 | 1.409 | C23-C24-C25 | 119.337 |
| C22-C23 | 1.402 | C24-C25-C26 | 119.558 |
| C23-C24 | 1.407 | C25-C26-C27 | 121.091 |
| C24-C25 | 1.410 | C26-C27-C22 | 119.221 |
| C25-C26 | 1.393 | C24-C28-C29 | 179.882 |
| C26-C27 | 1.396 | C7-O17-C18 | 119.420 |
| C27-C22 | 1.407 | O17-C18-C19 | 107.159 |
| C24-C28 | 1.43 | C18-C19-O20 | 107.243 |
| C28-C29 | 1.21 | C19-O20-C21 | 114.021 |
| C7-O17 | 1.355 | C8-O12-C13 | 120.712 |
| O17-C18 | 1.416 | O12-C13-C14 | 106.827 |
| C18-C19 | 1.518 | C13-C14-O15 | 107.491 |
| C19-O20 | 1.401 | C14-O15-C16 | 113.961 |
| O20-C21 | 1.404 |  |  |
| C8-O12 | 1.355 |  |  |
| O12-C13 | 1.427 |  |  |
| C13-C14 | 1.519 |  |  |
| C14-O15 | 1.404 |  |  |
| O15-C16 | 1.40 |  |  |

**Table S7**: Optimized structural parameters of Gefitinib at B3LYP/SVP

| **Bond** | **Bond length (Å)** | **Bond** | **Bond angle (◦)** |
| --- | --- | --- | --- |
| N1-C2 | 1.314 | N1-C2-N2 | 127.399 |
| C3-N3 | 1.348 | C2-N3-C4 | 117.786 |
| N3-C4 | 1.327 | N3-C4-C5 | 121.121 |
| C4-C5 | 1.436 | C4-C5-C10 | 115.228 |
| C5-C10 | 1.429 | C5-C10-N1 | 121.966 |
| C10-N1 | 1.363 | C10-N1-C2 | 116.168 |
| C5-C6 | 1.420 | C5-C6-C7 | 121.257 |
| C6-C7 | 1.377 | C6-C7-C8 | 119.897 |
| C7-C8 | 1.440 | C7-C8-C9 | 119.732 |
| C8-C9 | 1.383 | C8-C9-C10 | 120.830 |
| C9-C10 | 1.421 | C9-C10-C5 | 119.320 |
| C4-N11 | 1.383 | C10-C5-C6 | 118.853 |
| N11-C24 | 1.411 | C24-C25-C26 | 120.550 |
| C24-C25 | 1.404 | C25-C26-C27 | 119.710 |
| C25-C26 | 1.396 | C26-C27-C28 | 120.052 |
| C26-C27 | 1.401 | C27-C28-C29 | 120.345 |
| C27-C28 | 1.393 | C28-C29-C24 | 120.196 |
| C28-C29 | 1.394 | C29-C24-C25 | 119.138 |
| C29-C24 | 1.406 | N16-C17-C18 | 112.687 |
| C7-O12 | 1.361 | C17-C18-O19 | 110.622 |
| 012-C13 | 1.429 | C18-O19-C20 | 112.131 |
| C13-C14 | 1.518 | O19-C20-C21 | 110.911 |
| C14-C15 | 1.530 | C20-C21-N16 | 112.564 |
| CQ5-N16 | 1.459 | C21-N16-C17 | 109.596 |
| N16-C17 | 1.462 | C7-O12-C13 | 116.988 |
| C17-C18 | 1.532 | O12-C13-C14 | 107.876 |
| C18-O19 | 1.417 | C13-C14-C15 | 112.123 |
| O19-C20 | 1.417 | C14-C15-N16 | 112.678 |
| C20-C21 | 1.532 |  |  |
| C21-N16 | 1.465 |  |  |

**Reported inhibitory Potential against various cell lines**

According to reported literatures;

<https://doi.org/10.1038/nbt1068>,

<https://www.nature.com/articles/nbt.1990> &

<https://www.nature.com/articles/nbt1358>,

**There is insufficient evidence that Alectinib is selective and potent inhibitor of NEK kinases. In addition, the activity of drugs against certain kinases was evaluated at a concentration of 10 µM. The total time period was not found in the available literature. However, according to ATTC rules, the compounds are termed anticancer if they inhibit the maximal growth decrease of respective cells during a 72-hour period. The absence of data in the table indicates that compounds have a poor potential to inhibit cell growth at the concentration and time period examined (see table below, no activity found against NEK7)**.

| **NEK kinase** | **ABT-869** | **AMG-706** | **AST-487** | **AZD-1152HQPA** | **BIRB-796** | **BMS-387032/SNS 032** | **CHIR-258/TKI-258** | **CHIR-265/RA** |
| --- | --- | --- | --- | --- | --- | --- | --- | --- |
| **NEK1** |  |  |  |  |  |  |  |  |
| **NEK2** |  |  |  |  |  |  | **2000** |  |
| **NEK6** |  |  |  |  |  |  | **2300** | **3200** |
| **NEK5** |  |  | **1100** |  |  |  | **1500** |  |
| **NEK7** |  |  |  |  |  |  | **5600** |  |
| **NEK9** |  |  |  |  |  |  | **9100** |  |

We are currently working on NIMA-related kinases. These kinases are extremely underexplored therapeutic targets for anti-cancer drug discovery and development. Regarding selective and possible inhibition of NEK kinases, there is a paucity of information. Among the eleven members of this family, only NEK2 and NEK5 have been widely researched, whereas NEK7 remains an unexplored target for the development of anti-cancer drugs. It is involved in the activation of the NLRP3 inflammasone signaling pathway **(doi: 10.1038/s41586-019-1295-z),** cytokinesis, spindle assembly **(doi: 10.4161/15384101.2014.994949),** and other functions. Any abnormal expression resulted in not only cancer but also severe effects such as brain diseases, bone disorders, and dyslipidemias, etc., thus we are developing selective and powerful inhibitors of NEK kinases, particularly NEK7. Due to the structural similarities between NEK and other kinases, it is possible that Alectinib and other FDA-approved medications might also target the NEK kinase family. In our future research, we want to construct a pharmacophore model for a top-ranked FDA medicine acquired from current research in order to synthesize novel chemical moieties.

In addition, we have determined the selectivity index from the reported literature which is showing that none of reported inhibitors is selective against NEK7 (see table below).

| **Compound** | **NEK7** | **ALK** | **Selectivity index for NEK7^a^** |
| --- | --- | --- | --- |
| BI-2536 | 6100 | 160 | 0.02 |
| AZD-2171 | --- | 2300 | -- |
| CHIR-258/TKI-258 | 5600 | 3500 | 0.625 |
| Staurosporine | 4500 | 32 | 0.007 |
| SU-14813 | 3200 | 490 | 0.153 |
| Sunitinib | 4100 | 170 | 0.041 |
| SB-431542 | -- | 750 | --- |
| ERLOTINIB | --- | 1200 | --- |
| EKB-569 | -- | 7100 | --- |
| Alectinib | 3880 (calculated in our study) | -- | 1 |

^a^Selectivity index defined as *IC*[ALK]/*IC*[NEK7]

However, after screening Alectinib against NEK7, we docked Alectinib with NEK2, although binding interactions and docking scores were insufficient for NEK2 inhibition. Therefore, it is strongly suggested that Alectinib might be a selective inhibitor of NEK7, and more in-vitro research is advised. In addition, 675 Alectinib derivatives from PubChem were obtained and subjected to virtual screening, MD simulation, and in-silico ADMET prediction. Only one compound (146476703) had higher docking scores and a more stable protein-ligand interactions than Alectinib. The primary objective of virtual screening is to obtain a starting point for the synthesis of Alectinib derivatives in our future research. We are very thankful in advance to the reviewer to understand our aim and rationale behind the work. Moreover, we are also thankful to the reviewer to consider incorporation of additional data as the justification of the study.
